# Quantitative susceptibility mapping in the brain reflects spatial expression of genes involved in iron homeostasis and myelination

**DOI:** 10.1002/hbm.26688

**Published:** 2024-06-19

**Authors:** Zoe Cohen, Laurance Lau, Maruf Ahmed, Clifford R. Jack, Chunlei Liu

**Affiliations:** ^1^ Department of Electrical Engineering and Computer Sciences University of California, Berkeley Berkeley California USA; ^2^ Mayo Foundation for Medical Education and Research Rochester Minnesota USA; ^3^ Helen Wills Neuroscience Institute University of California, Berkeley Berkeley California USA

**Keywords:** Allen human brain atlas, iron, myelination, QSM

## Abstract

Quantitative susceptibility mapping (QSM) is an MRI modality used to non‐invasively measure iron content in the brain. Iron exhibits a specific anatomically varying pattern of accumulation in the brain across individuals. The highest regions of accumulation are the deep grey nuclei, where iron is stored in paramagnetic molecule ferritin. This form of iron is considered to be what largely contributes to the signal measured by QSM in the deep grey nuclei. It is also known that QSM is affected by diamagnetic myelin contents. Here, we investigate spatial gene expression of iron and myelin related genes, as measured by the Allen Human Brain Atlas, in relation to QSM images of age‐matched subjects. We performed multiple linear regressions between gene expression and the average QSM signal within 34 distinct deep grey nuclei regions. Our results show a positive correlation (*p* < .05, corrected) between expression of ferritin and the QSM signal in deep grey nuclei regions. We repeated the analysis for other genes that encode proteins thought to be involved in the transport and storage of iron in the brain, as well as myelination. In addition to ferritin, our findings demonstrate a positive correlation (*p* < .05, corrected) between the expression of ferroportin, transferrin, divalent metal transporter 1, several gene markers of myelinating oligodendrocytes, and the QSM signal in deep grey nuclei regions. Our results suggest that the QSM signal reflects both the storage and active transport of iron in the deep grey nuclei regions of the brain.

## Graphical abstract

We compared values from quantitative susceptibility mapping (QSM) images and spatial gene expression, measured by the Allen Human Brain Atlas. Our findings demonstrate a positive correlation between the expression of ferritin, ferroportin, transferrin, several gene markers of myelinating oligodendrocytes, and QSM in deep grey nuclei regions.

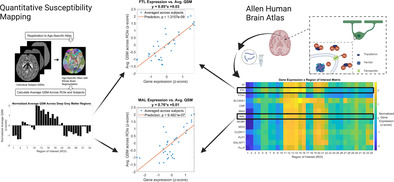

## 1 INTRODUCTION

In the brain, iron is required in large amounts for proper functioning, including for neurotransmitter synthesis and signaling, ATP production, and especially for the myelination of axons, which provides insulation for the transmission of neural signals (Connor & Menzies, 1996; Ortiz et al., 2004; Sheftel et al., 2012). Gene expression can serve as a window into the underlying proteins involved in these processes (Hawrylycz et al., 2012). The overall mechanism of iron homeostasis can also be observed by looking at the anatomically varying pattern that iron follows in the brain. In normal aging, iron accumulates in deep grey matter areas due to a highly regulated, robust process that has evolved to control its storage, release, and transport in the brain (Bradbury, 1997; Ke & Ming Qian, 2007; Moos et al., 2007). Although the complete mechanism of iron homeostasis in the brain is not known, the trajectory of iron accumulation in the brain over the lifespan has been well observed using Quantitative Susceptibility Mapping (QSM) MRI (Li et al., 2014; Möller et al., 2019; Zhang et al., 2018, 2019). QSM has been instrumental in identifying the importance of this specific pattern for normal brain functioning (Carpenter et al., 2016; He et al., 2015; Li et al., 2015).

QSM is an MRI modality in which the signal of each voxel in the image is a measurement of the corresponding magnetic susceptibility, the ratio between the magnetization of a material when placed in an external magnetic field and the strength of the applied magnetic field, within that voxel (de Rochefort et al., 2010; Liu et al., 2012; Liu, Li, et al., 2015; Wang & Liu, 2015; Wei et al., 2016). In addition to being non‐invasive and relatively high spatial resolution, QSM yields quantitative information about the susceptibility of tissues, which reflects their molecular composition (Deistung et al., 2008; Li et al., 2011; Liu, 2010; Liu, Wei, et al., 2015; Wharton et al., 2010). Iron and myelin are believed to be the two primary sources of susceptibility contrast observed in the brain (Langkammer et al., 2012; Li et al., 2014; Möller et al., 2019). Ferritin (Ft), the iron storage molecule, is paramagnetic and dominates the positive QSM signal in regions that accumulate iron (Langkammer et al., 2012; Liu, Wei, et al., 2015; Wu et al., 2012; Zheng et al., 2013). The molecular basis behind the positive susceptibility is Ft's large iron core, which can hold up to 4500 iron molecules (Theil, 1987). In addition to its storage capacity, there is evidence that the heavy‐chain subunit of Ft (H‐Ft) can transport iron, and a receptor that binds H‐Ft has been identified on myelinating oligodendrocytes (Chiou et al., 2018; Todorich et al., 2011).

Ferritin, however, is not the only molecule responsible for delivering iron to the deep grey nuclei. Other prominent players include transferrin (Tf), transferrin receptor (TFR), divalent metal transporter 1 (DMT1), and ferroportin (Fpn) (Mills et al., 2010; Moos et al., 2007). Tf and TFR are thought to provide the primary route of iron influx into the brain across the blood brain barrier (BBB) (Bradbury, 1997; Ke & Ming Qian, 2007; Moos et al., 2007). TFR is present on the luminal side of the brain microvascular endothelial cells (BMECs), which make up the BBB, and iron is taken up by these cells via receptor‐mediated endocytosis of Tf‐bound TFR (Moos et al., 2007; Skjørringe et al., 2015). Separation from the Tf‐TFR complex is facilitated by the acidic environment of the endosome, which then releases the non‐Tf bound iron into the BMEC cytosol via DMT1 (Skjørringe et al., 2015). Afterwards, iron is ultimately transported into the brain by Fpn, the only known iron exporter (Moos et al., 2007; Wu et al., 2004). In addition to BMECs, Fpn is expressed in glial cells like astrocytes and oligodendrocytes, as well as neurons (Qian & Ke, 2019; Wu et al., 2004). Upon release from BMECs, non‐Tf bound iron may be taken up by astrocytes before being distributed to neurons and other cells (Qian & Ke, 2019). This is supported by the presence of DMT1, which can transport iron and other metal ions across cell membranes, on the end feet of astrocytes (Cheli et al., 2020; Skjørringe et al., 2015). These pathways formed by the proteins Tf, TFR, DMT1, Fpn, and Ft, are thought to make up the main mechanism of iron transport into brain regions, as well as storage.

Besides iron‐loaded Ft, the other main contribution to the QSM signal in the brain is myelin, which is diamagnetic and results in negative QSM in the white matter (Langkammer et al., 2012; Li et al., 2014; Liu et al., 2010; Zhang et al., 2019). Various genes have been implicated in the process of myelination, most of which are expressed by oligodendrocytes (Chavarria‐Siles et al., 2015). Oligodendrocytes are glial cells that wrap around the axons of neurons, forming an insulating sheath (Baumann & Pham‐Dinh, 2001). Myelination is initiated by changes in gene expression, which prompt oligodendrocyte precursor cells to migrate within the brain, branch morphologically, and form sheaths around axons (Baumann & Pham‐Dinh, 2001; Kuhn et al., 2019). (Chavarria‐Siles et al., 2015) define a set of genes involved in this process, including myelin basic protein (MBP), 2′,3′‐cyclic nucleotide 3′‐phosphodiesterase (CNP), myelin‐associated glycoprotein (MAG), myelin and lymphocyte protein (MAL), myelin‐associated oligodendrocytic basic protein (MOBP), myelin oligodendrocyte glycoprotein (MOG), claudin‐11 (CLDN11), proteolipid protein (PLP1), galactose‐3‐O‐sulfotransferase‐1 (GAL3ST1), proteolipid plasmolipin (PLLP), integrin‐linked kinase (ILK), oligodendrocyte‐myelin glycoprotein (OMG), kallikrein‐related peptidase 6 (KLK6), oligodendrocyte transcription factor 2 (OLIG2), eukaryotic translation initiation factor 2 alpha kinase 3 (EIF2AK3), POU domain class 3 transcription factor 1 (POU3F1) and neuregulin 1 (NRG1). All of these are expressed by oligodendrocytes, except NRG1 which is expressed in neurons and astrocytes but acts to regulate the migration of oligodendrocyte precursor cells (Ortega et al., 2012). These genes are implicated in the production and reorganization of the lipids and proteins that compose the myelin sheath, many of which are diamagnetic (Duyn et al., 2007).

Although the QSM image is captured at millimeter resolution, we wanted to assess whether the susceptibility signal from QSM could reflect iron homeostasis on a molecular level, as measured by gene expression. This question has already been supported by other studies, particularly (Wang et al., 2022), which concludes that the expression of genes involved in iron homeostasis and myelination is correlated with the QSM signal in the deep grey nuclei. Other previous genome‐wide association studies have shown a correlation between biomarkers of body iron levels and genes involved in iron transport and storage in the brain (Benyamin et al., 2014; Elliott et al., 2018). However, these analyses find correlations across subjects and are limited by a single measure of expression for a given gene that is not localized to the brain but in the blood, therefore neglecting the spatial dimension of gene expression. For this reason, we use the Allen Human Brain Atlas (AHBA), a publicly available dataset of gene expression measured across regions of the brain, to expand upon these studies in characterizing the relationship between QSM and brain iron homeostasis (Allen Institute for Brain Science, 2010; Hawrylycz et al., 2012).

We conduct a comparative analysis between QSM and expression of genes known to be involved in iron transport and myelination across brain regions, with a goal of further understanding the pathways by which iron selectively accumulates in deep grey nuclei regions of the aging brain. We found that QSM is related to the expression of genes relevant to iron homeostasis and myelination measured across these deep grey nuclei regions.

## 2 METHODS

We performed linear regressions between age‐matched QSM and gene expression data across functionally distinct regions in the deep grey matter. A linear regression model was fit for each of 15,627 unique genes from the AHBA microarray dataset, collected from six normal post‐mortem brains, aged 24–57 (median 44), both male and female (Allen Institute for Brain Science, 2010; Hawrylycz et al., 2012; Shen et al., 2012), and the average QSM of nine healthy subjects, aged 41–49 (median 45), both male and female, calculated across the same regions (Zhang et al., 2018). In order to test the robustness of our result, we repeated the linear regression analysis using a second set of QSM images collected from 10 healthy subjects, aged 41–49 (median 45), both male and female (Cogswell et al., 2021). More information about these datasets, as well as processing and the set‐up of the regression problem, are described as follows.

### 2.1 MRI acquisition and post processing

We calculated the average QSM signal in 34 distinct regions in the brain for nine healthy subjects age‐matched to the median age of the AHBA dataset. The QSM dataset is described in (Zhang et al., 2018). Briefly, subjects were scanned on a 3T scanner (GE Healthcare Signa HDxt at Rui Jin Hospital in Shanghai, China) following approval of the institutional review board and signing of informed consent. T2*‐weighted images were acquired using a three‐dimensional multi‐echo gradient echo sequence (TE1/spacing/TE8 = 5.468/3/26.5 ms, TR = 54.6 ms, original spatial resolution .86 × .86 × 2 mm^3^ resampled to 1 × 1 × 1 mm^3^). The QSM image was then reconstructed using STI Suite V3.0 (https://chunleiliulab.github.io/software.html). The brain was extracted from each image using FSL's brain extraction tool (BET) (Smith et al., 2004). Following Laplacian‐based phase unwrapping and normalization, background phase removal was then performed using a variable‐kernel Sophisticated Harmonic Artifact Reduction for Phase data (V‐SHARP) method (Schweser et al., 2010; Wu et al., 2012; Zhu & Schofield, 2003). The radius of the spherical mean value filter varied from 1 pixel at the boundary of the brain to 25 pixels around the center of the brain (Wu et al., 2012). Lastly, the STAR‐QSM algorithm was used to reconstruct the QSM image from the filtered phase image (Wei et al., 2015). The resulting susceptibility values were referenced relative to the mean susceptibility across the whole brain, which has been shown to be as reliable as using CSF as the susceptibility reference (Li et al., 2014).

**FIGURE 1:**
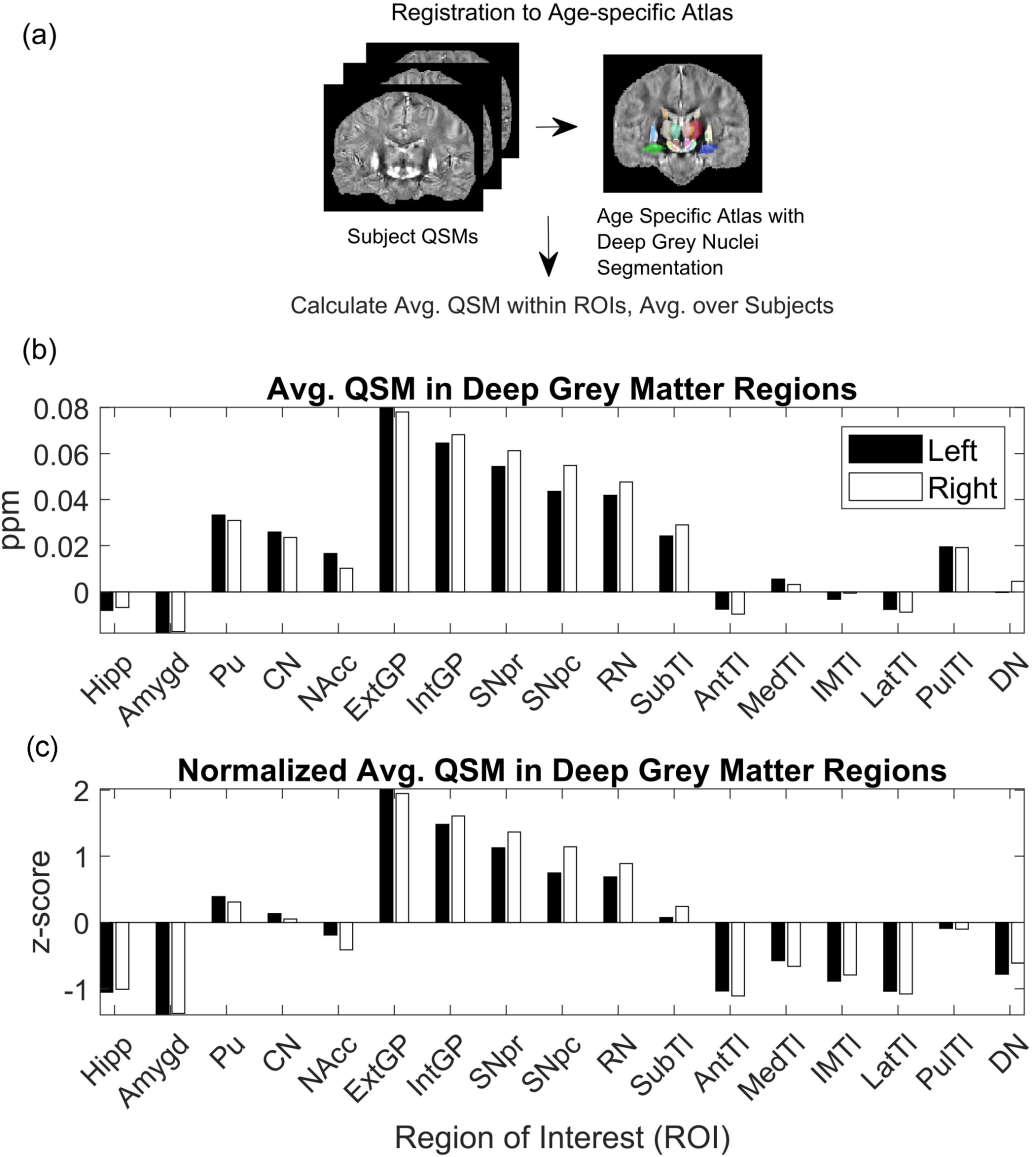
Group QSM analysis pipeline. (a) QSM registration and preprocessing. Advanced Normalization Tools (ANTs) was used to register nine healthy individual QSM brains, aged 41‐49, to the age‐specific QSM atlas constructed in (Zhang et al., 2018). Following registration, the segmentation associated with the atlas was used to calculate average QSM over all subjects across regions in the deep grey nuclei. (b) Average QSM across deep grey nuclei regions (ppm). (c) Normalized average QSM across deep grey nuclei regions (z‐score). Regions of interest on the x‐axis correspond to Left (L) and Right (R) Hippocampus (Hipp), Amygdala (Amygd), Putamen (Pu), Caudate Nucleus (CN), Nucleus Accumbens (NAcc), External Globus Pallidus (ExtGP), Internal Globus Pallidus (IntGP), Substantia Nigra pars reticulata (SNpr), Substantia Nigra pars compacta (SNpc), Red Nucleus (RN), Subthalamic nuclei (SubTl), Anterior nuclei of the Thalamus (AntTl), Median nuclei of the Thalamus (MedTl), Intermedullary nuclei of the Thalamus (IMTl), Lateral nuclei of the Thalamus (LatTl), Pulvinar nuclei of the Thalamus (PulTl), and Dentate Nucleus (DN).

We used Advanced Normalization Tools (ANTs) (Avants et al., 2009) to perform nonlinear registration between each individual QSM and the age‐specific QSM atlas constructed using group‐wise registration in (Zhang et al., 2018). The inverse transform generated from the registration was used to warp the segmentation from atlas space back to subject space. The mean signal for each region of interest (ROI) was then calculated by applying the warped mask to the original QSM subject image and averaging all non‐zero voxels. This analysis was repeated for all nine subjects, and the mean QSM values across subjects were then normalized using the z‐score, yielding the distribution shown in Figure 1. The labeled ROIs are also listed in Table 1.

**TABLE 1:** Labels for regions of interest (ROIs) in the deep grey nuclei used in our analysis.

ROI	QSM atlas ROI	Corresponding AHBA ROI(s)
1	Hippocampus (left)	Dentate Gyrus (left), CA1 Field (left), CA2 Field (left), CA3 Field (left), CA4 Field (left), Subiculum (left)
2	Hippocampus (right)	Dentate Gyrus (right), CA1 Field (right), CA2 Field (right), CA3 Field (right), CA4 Field (right), Subiculum (right)
3	Amygdala (left)	Amygdalohippocampal Transition Zone (left), Basolateral Nucleus (left), Basomedial Nucleus (left), Central Nucleus (left), Cortico‐medial Group (left), Lateral Nucleus (left)
4	Amygdala (right)	Amygdalohippocampal Transition Zone (right), Basolateral Nucleus (right), Basomedial Nucleus (right), Central Nucleus (right), Cortico‐medial Group (right), Lateral Nucleus (right)
5	Putamen (left)	Putamen (left)
6	Putamen (right)	Putamen (right)
7	Caudate Nucleus (left)	Body of Caudate Nucleus (left), Head of Caudate Nucleus (left), Tail of Caudate Nucleus (left)
8	Caudate Nucleus (right)	Body of Caudate Nucleus (right), Head of Caudate Nucleus (right), Tail of Caudate Nucleus (right)
9	Nucleus Accumbens (left)	Nucleus Accumbens (left)
10	Nucleus Accumbens (right)	Nucleus Accumbens (right)
11	External Globus Pallidus (left)	Globus Pallidus, External Segment (left)
12	External Globus Pallidus (right)	Globus Pallidus, External Segment (right)
13	Internal Globus Pallidus (left)	Globus Pallidus, Internal Segment (left)
14	Internal Globus Pallidus (right)	Globus Pallidus, Internal Segment (right)
15	Pars Reticulata of Substantia Nigra (left)	Substantia Nigra, Pars Reticulata (left)
16	Pars Reticulate of Substantia Nigra (right)	Substantia Nigra, Pars Reticulata (right)
17	Pars Compacta of Substantia Nigra (left)	Substantia Nigra, Pars Compacta (left)
18	Pars Compacta of Substantia Nigra (right)	Substantia Nigra, Pars Compacta (right)
19	Red Nucleus (left)	Red Nucleus (left)
20	Red Nucleus (right)	Red Nucleus (right)
21	Subthalamic Nucleus (left)	Subthalamic Nucleus (left)
22	Subthalamic Nucleus (right)	Subthalamic Nucleus (right)
23	Anterior Nuclei of Thalamus (left)	Anterior Group of Nuclei (left)
24	Anterior Nuclei of Thalamus (right)	Anterior Group of Nuclei (right)
25	Median Nuclei of Thalamus (left)	Medial Group of Nuclei (left)
26	Median Nuclei of Thalamus (right)	Medial Group of Nuclei (right)
27	Internal Medullary Lamina of Thalmus (left)	Caudal Group of Intralaminar Nuclei (left), Rostral Group of Intralaminar Nuclei (left)
28	Internal Medullary Lamina of Thalmus (right)	Caudal Group of Intralaminar Nuclei (right), Rostral Group of Intralaminar Nuclei (right)
29	Lateral Nuclei of Thalamus (left)	Lateral Group of Nuclei (left), Medial Geniculate Complex (left)
30	Lateral Nuclei of Thalamus (right)	Lateral Group of Nuclei (right), Medial Geniculate Complex (right)
31	Pulvinar Nuclei of Thalamus (left)	Lateral Group of Nuclei (left), Posterior Group of Nuclei (left)
32	Pulvinar Nuclei of Thalamus (right)	Lateral Group of Nuclei (right), Posterior Group of Nuclei (right)
33	Dentate Nucleus (left)	Dentate Nucleus (left)
34	Dentate Nucleus (right)	Dentate Nucleus (right)

*Note*: The second column corresponds to regions from Zhang's QSM atlas segmentation (Zhang et al., 2018), and the third column corresponds to regions from the Allen Human Brain Atlas (AHBA) (Allen Institute for Brain Science, 2010; Hawrylycz et al., 2012; Shen et al., 2012). Most ROIs from the two segmentations matched one‐to‐one, however there were some cases in which ROIs in the AHBA corresponded to subdivisions of an ROI from Zhang's segmentation. In these instances, all sub‐regions are reported in the same row of the ROI which contains them.

**FIGURE 2:**
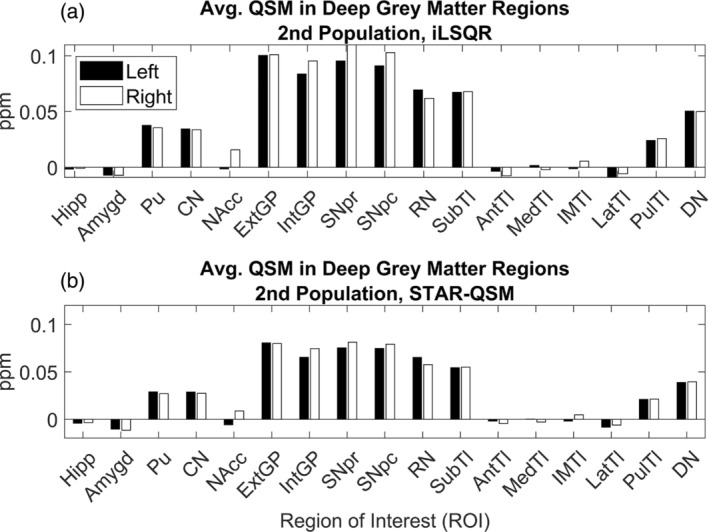
Average QSM in Deep Grey Matter Regions, 2nd Population. (a) iLSQR reconstruction. (b) STAR‐QSM reconstruction. Regions of interest on the x‐axis correspond to Left (L) and Right (R) Hippocampus (Hipp), Amygdala (Amygd), Putamen (Pu), Caudate Nucleus (CN), Nucleus Accumbens (NAcc), External Globus Pallidus (ExtGP), Internal Globus Pallidus (IntGP), Substantia Nigra pars reticulata (SNpr), Substantia Nigra pars compacta (SNpc), Red Nucleus (RN), Subthalamic nuclei (SubTl), Anterior nuclei of the Thalamus (AntTl), Median nuclei of the Thalamus (MedTl), Intermedullary nuclei of the Thalamus (IMTl), Lateral nuclei of the Thalamus (LatTl), Pulvinar nuclei of the Thalamus (PulTl), and Dentate Nucleus (DN).

The collection of the second dataset is described in (Cogswell et al., 2021). Subjects were scanned on a 3T scanner (Siemens Prisma VE11C at Mayo Clinic) as part of the Mayo Clinic Study of Aging, which had the approval of the institutional review board and written informed consent from participants. Images were acquired using a three‐dimensional multi‐echo gradient echo sequence (TE1/spacing/TE5 = 6.7/3.9/22.4 ms, TR = 28 ms, original spatial resolution .52 × .52 × 1 mm^3^ resampled to 1 × 1 × 1.8 mm^3^), and STI Suite was used to reconstruct the QSM for each subject. The brain mask was generated using FSL's BET. Masking, Laplacian‐based phase unwrapping and background phase removal were performed using V‐SHARP (Schweser et al., 2010; Smith et al., 2004; Wu et al., 2012; Zhu & Schofield, 2003). The radius of the spherical mean value filter was set to 1 pixel at the boundary of the brain and 12 pixels at the center of the brain. Finally, QSM images were calculated from the filtered phase using both the STAR‐QSM and Improved Sparse Linear Equation and Least‐squares (iLSQR) algorithms (Li et al., 2015; Wei et al., 2015). Susceptibility values were again referenced relative to the mean susceptibility across the whole brain. Normalized average QSM values were calculated in ROIs following the same processing pipeline for the first dataset. The non‐normalized values for iLSQR and STAR‐QSM are plotted in Figure 2.

### 2.2 DNA microarray survey and post processing

The AHBA is a microarray profile of gene expression values collected from the autopsied normal brains of six individuals, median age 44 (Allen Institute for Brain Science, 2010; Hawrylycz et al., 2012; Shen et al., 2012). Before inclusion in the dataset, the brain tissue and case profile of each individual was subjected to various screening and evaluation, in order to ensure the integrity of the mRNA and the validity of using the individual as a normal control, as described in (Allen Human Brain Atlas Technical White Paper: Case Qualification and Donor Profiles, 2013). Dissection of the brains into regions for subsequent processing and microarray analysis resulted in 58,692 gene expression measurements for each of 3702 distinct tissue samples across the brain (Allen Human Brain Atlas Technical White Paper: Microarray Survey, 2013).

**FIGURE 3:**
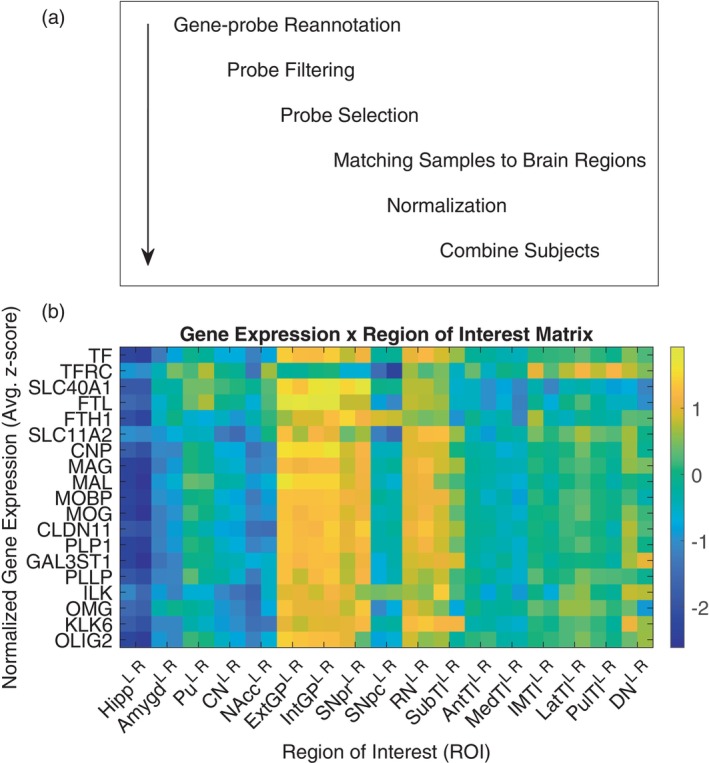
Gene expression analysis pipeline. (a) Processing pipeline for Allen Human Brain Atlas (AHBA) dataset. We used software developed in (Arnatkevic̆iūtė et al., 2019) for gene‐probe reannotation, probe filtering, and probe selection of the AHBA dataset (Allen Institute for Brain Science, 2010; Hawrylycz et al., 2012; Shen et al., 2012). We filtered the probes to exclude those with expression levels at or below background levels in 50% or more of all samples. The probe with the highest differential stability across subjects was then selected. AHBA sampled regions were matched to the corresponding ROIs in the QSM atlas segmentation (Zhang et al., 2018). (b) Expression of genes involved in iron homeostasis and myelination across deep nuclei regions. The gene expression by ROI matrix depicts expression of a subset of genes across these ROIs, following normalization and averaging across subjects. Regions of interest on the x‐axis correspond to Left (L) and Right (R) Hippocampus (Hipp), Amygdala (Amygd), Putamen (Pu), Caudate Nucleus (CN), Nucleus Accumbens (NAcc), External Globus Pallidus (ExtGP), Internal Globus Pallidus (IntGP), Substantia Nigra pars reticulata (SNpr), Substantia Nigra pars compacta (SNpc), Red Nucleus (RN), Subthalamic nuclei (SubTl), Anterior nuclei of the Thalamus (AntTl), Median nuclei of the Thalamus (MedTl), Intermedullary nuclei of the Thalamus (IMTl), Lateral nuclei of the Thalamus (LatTl), Pulvinar nuclei of the Thalamus (PulTl), and Dentate Nucleus (DN).

The processing pipeline in Figure 3 describes the method of preparation for the gene expression dataset, before it could be used in the regression analysis. This procedure largely follows that described in (Arnatkevic̆iūtė et al., 2019), which provides a thorough evaluation of the methods chosen for each step and the software used to execute these methods. We used Arnatkevic̆iūtė's software package (Arnatkevic̆iūtė et al., 2019) to perform gene‐probe reannotation, probe filtering, and probe selection across all probes and all subjects. We relied on the probe‐gene reannotation done by (Arnatkevic̆iūtė et al., 2019) using the National Center for Biotechnology Information (NCBI) human reference genome in March 2018 (Genome Reference Consortium Human Build 38, 2013). We excluded probes with expression levels at or below background levels in at least 50% of all samples (this information is provided by AHBA for each probe). Of those remaining, we chose a representative probe for each gene by selecting the one with the most consistent pattern of expression across all six brains, as measured by differential stability (Hawrylycz et al., 2015).

Next, we matched samples within deep grey matter regions in the AHBA segmentation to the same regions defined by QSM‐based brain atlases (Li et al., 2019; Zhang et al., 2018). Specifically, we chose Zhang's QSM age‐specific atlas segmentation (Zhang et al., 2018). We chose to match samples by region name, rather than using the provided MNI coordinates of samples, as we focus only on deep grey regions which mostly map one‐to‐one with regions sampled in the AHBA. Additionally, we found that the MNI coordinates reported by AHBA for sampled regions did not match up to accurate regions in our segmentation, likely due to registration discrepancies in the mapping between subject and MNI space. Some regions in Zhang's atlas segmentation, like the Hippocampus and Amygdala, were found to be subdivided into multiple smaller regions in the AHBA ontology^1^ (see Figure S5). In these cases, we averaged across all smaller regions within the ROI in Zhang's segmentation which encompassed them. Table 1 summarizes the results of our assignment between AHBA sample regions and ROIs in Zhang's segmentation.

Finally, normalization was performed for all genes across all matched samples using z‐score. This method was chosen due to its simplicity and demonstrated ability to minimize donor‐specific effects when normalizing for each subject separately (Arnatkevic̆iūtė et al., 2019). These z‐scores were then averaged across subjects to yield a single set of normalized gene expression values across ROIs defined in Zhang's segmentation. We report the correlation coefficients calculated between the normalized gene expression of all iron and myelination genes considered in Figure S4.

### 2.3 Linear regression analysis

Following pre‐processing using the methods of (Arnatkevic̆iūtė et al., 2019) as described above, we were left with a set of 15,627 gene expression vectors that measure the expression level of a unique gene averaged across all AHBA subjects, for each ROI defined in Zhang's segmentation. Let $x_{i}$ denote the vector of normalized relative expression of gene *i*, averaged across all six subjects (avg. z‐score), for all deep gray matter ROIs. Element *j* of $x_{i}$ is the averaged z‐score of gene *i* measured for ROI *j*, j=1,…,J, where J is the total number of ROIs. We define y as the vector of normalized QSM (z‐score) averaged across nine separate subjects. Element *j* of y, denoted $y_{j}$, is the averaged QSM z‐score across ROI *j*, j=1,…,J. For each gene *i* (from 1 to 15,627), we solved for the vector $\beta_{i}={\left[\beta_{0,i},\;\beta_{1,i}\right]}^{T}$ that satisfies the following linear regression problem:

(1) $$\;y={\left[\begin{array}{ccc} 1 & 1 & \cdots \\ x_{1,i} & x_{2,i} & \cdots \end{array}\;\begin{array}{c} 1\\ x_{J,i} \end{array}\right]}^{T}\left[\begin{array}{c} \beta_{0,i}\\ \beta_{1,i} \end{array}\right]=\left[\begin{array}{cc} 1 & x_{i} \end{array}\right]\beta_{i}=X_{i}\beta_{i}$$

After performing all 15,627 linear regressions, we calculated the *p* value of the estimated slope using a two‐tailed *t*‐test. We then applied multi‐comparison correction to the *p* values using the Benjamini–Hochberg procedure (Benjamini & Hochberg, 1995; Benjamini & Yekutieli, 2001; Reiner et al., 2003), which allows us to threshold *p* values by setting an upper bound on the false discovery rate (FDR), or the probability of falsely rejecting the null hypothesis. We chose FDR < .05, which is commonly used in microarray studies. We justify this method of multi‐comparison correction in our case, since we are interested in identifying genes that potentially have a relationship to the QSM signal, and this choice provides vastly more statistical power than the more conservative Bonferroni correction, which instead controls the family‐wise error rate (the probability of having at least one false positive). However, we also report results using the Bonferroni correction in order to highlight the most significant regression models. Since the QSM signal is known to be largely influenced from paramagnetic and diamagnetic species like iron‐loaded ferritin and myelin, we chose to focus on only the results of the regression for iron and myelination related genes (comprising a set of 23 genes identified from literature and listed in Tables S1 and S2).

## 3 RESULTS

### 3.1 Linear regression with iron transport and storage genes

**FIGURE 4:**
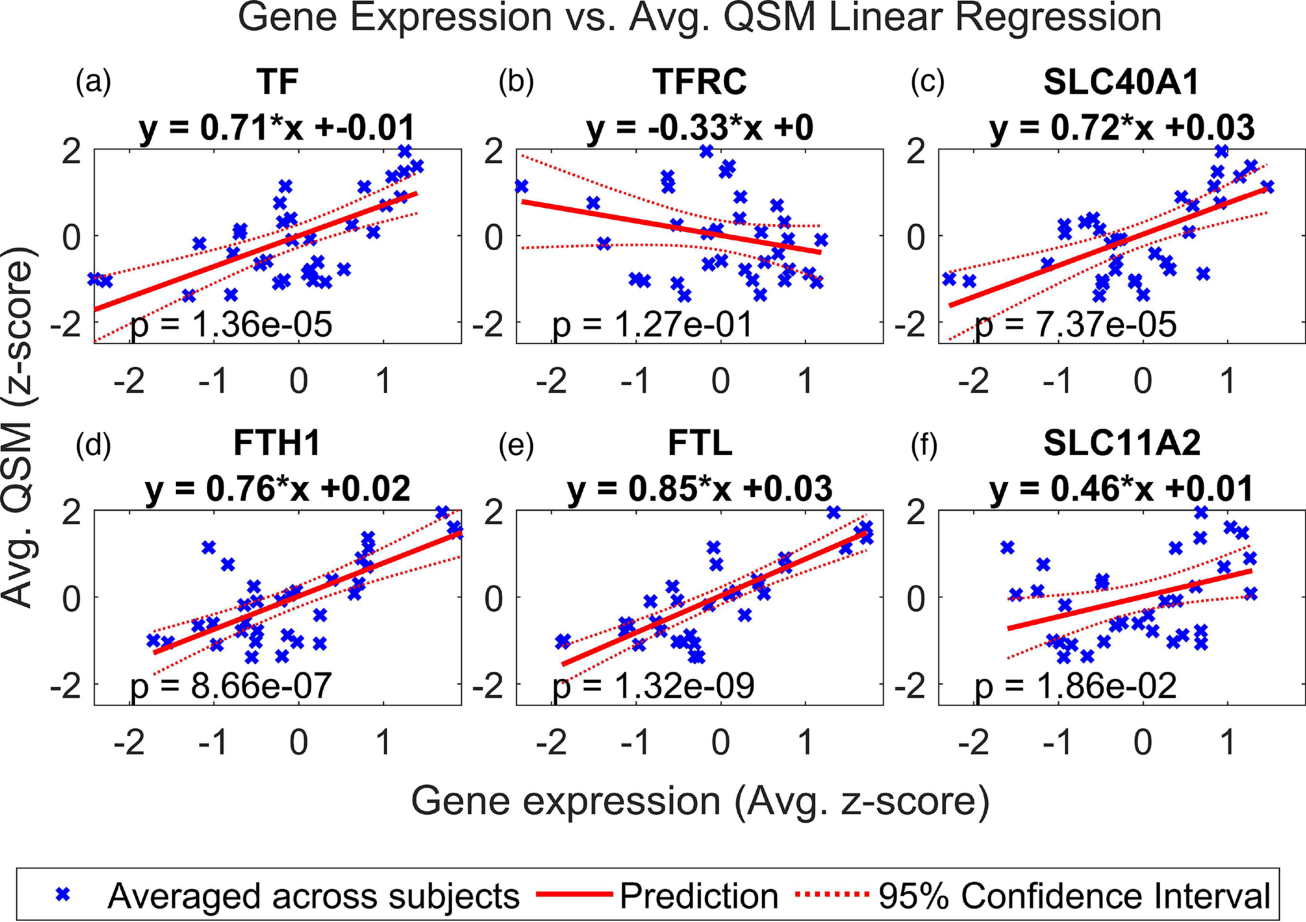
Multiple regression of QSM vs. iron related genes. Linear regression of QSM vs. normalized expression of (a) TF, (b) TFRC, (c) SLC40A1, (d) FTH1, (e) FTL, and (f) SLC11A2 in the deep grey nuclei regions. These refer to transferrin (TF), transferrin receptor (TFRC), ferroportin (SLC40A1), ferritin heavy chain (FTH1), ferritin light chain (FTL), and divalent metal transporter 1 (SLC11A2). QSM and gene expression were averaged across subjects. Regions of interest in the deep grey nuclei are listed in Table 1. See Figure S1 for the results of linear regression with the iron gene set performed for each subject separately.

We first focus on the results of the multiple linear regressions for genes encoding a set of proteins involved in iron homeostasis: Tf, TFR, Fpn, heavy‐chain Ft (H‐Ft), light‐chain Ft (L‐Ft), and DMT1. Figure 4a–f show the results of the linear regression using the first QSM dataset for each of the genes encoding these proteins, which are TF, TFRC, SLC40A1, FTH1, FTL, and SLC11A2, respectively. After conducting a two‐tailed *t*‐test (32 degrees of freedom), the regression models for Tf, H‐Ft, L‐Ft, Fpn, and DMT1 were found to be significant following the Benjamini–Hochberg procedure. Among those significant, the L‐Ft model had the smallest *p* value, 1.32e‐9, and then H‐ferritin with a *p* value of 8.66e‐7, and these remained significant following the Bonferroni correction. In all the significant cases, the correlation with the QSM signal is positive, and the value of the slope ranges from .46 to .85 (Figure 3). TFR did not yield a regression model with a significant *p* value following the Benjamini–Hochberg procedure, which is explained by the subject‐level analysis. TFR shows much higher variability in the slopes and *p* values across subjects, which ranged from 6.20e‐3 to 4.52e‐1 and −.53 to .31, respectively (Figure S1b). Conversely, Tf, H‐Ft, L‐Ft, Fpn, and DMT1 show good agreement in the linear regression on the subject level, with *p* values ranging from 4.53e‐5 to 1.81e‐2 for Tf, from 4.84e‐7 to 9.62e‐4 for H‐Ft, from 2.36e‐8 to 2.07e‐4 for L‐Ft, from 4.33e‐4 to 2.48e‐1 for Fpn, and from 3.43e‐2 to 1.93e‐1 for DMT1. Additionally, the slope of the regression line fitted for each subject was found to range from .58 to .69 for Tf, from .58 to .81 for H‐Ft, from .75 to .88 for L‐Ft, from .30 to .76 for Fpn, and from .23 to .52 for DMT1. The results of the subject‐level analysis are provided in Figure S1a–f.

### 3.2 Linear regression with myelination genes

**FIGURE 5:**
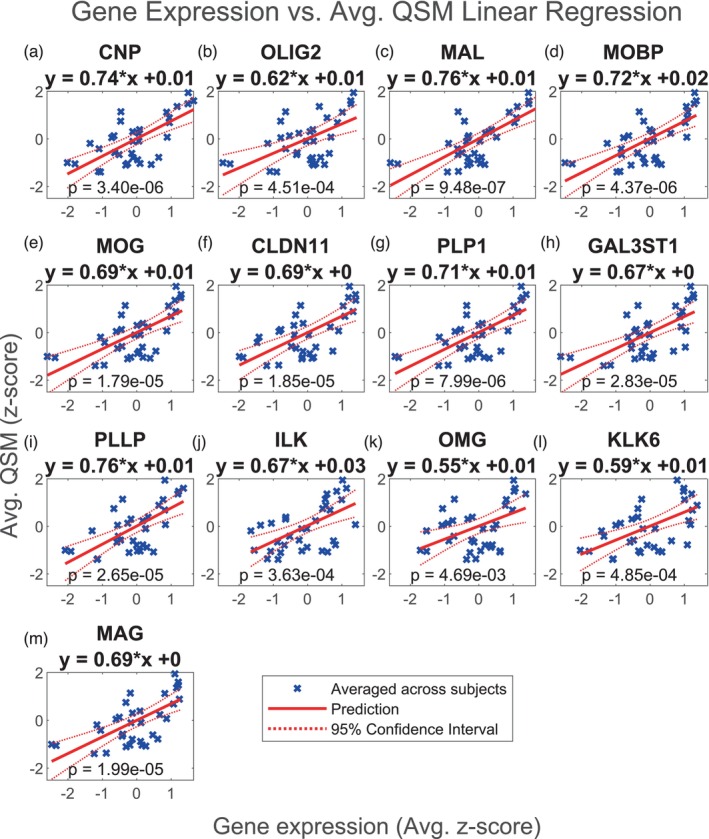
Multiple regression of QSM vs. myelin related genes. Linear regression of QSM vs. normalized expression of (a) CNP, (b) OLIG2, (c) MAL, (d) MOBP, (e) MOG, (f) CLDN11, (g) PLP1, (h) GAL3ST1, (i) PLLP, (j) ILK, (k) OMG, (l) KLK6 and (m) MAG in the deep grey nuclei regions. QSM and gene expression were averaged across subjects. Regions of interest in the deep grey nuclei are listed in Table 1. Only significant results are shown. These include 2′,3′‐cyclic nucleotide 3′‐phosphodiesterase (CNP), oligodendrocyte transcription factor 2 (OLIG2), myelin and lymphocyte protein (MAL), myelin‐associated oligodendrocytic basic protein (MOBP), myelin oligodendrocyte glycoprotein (MOG), claudin‐11 (CLDN11), proteolipid protein (PLP1), galactose‐3‐O‐sulfotransferase‐1 (GAL3ST1), proteolipid plasmolipin (PLLP), integrin‐linked kinase (ILK), oligodendrocyte‐myelin glycoprotein (OMG), kallikrein‐related peptidase 6 (KLK6) and myelin‐associated glycoprotein (MAG). See Figure S1 for the results of linear regression with the myelin gene set performed for each subject separately.

We also report the results of the multiple linear regressions for a set of 17 genes implicated in myelination: CLDN11, GAL3ST1, MAG, OMG, MBP, CNP, ILK, MAL, PLLP, NRG1, EIF2AK3, KLK6, PLP1, POU3F1, OLIG2, MOBP, MOG (Chavarria‐Siles et al., 2015). The proteins encoded by these genes are provided in Table S1. We again performed two‐tailed *t*‐tests (32 df) for each regression. Of these regression models, 13 were found to be significant following the Benjamini–Hochberg procedure, including CNP, OLIG2, MAL, MOBP, MOG, CLDN11, PLP1, GAL3ST1, PLLP, ILK, OMG, KLK6, and MAG as shown in Figures 5a–m. The MAL model had the smallest *p* value, 9.48e‐7, and then CNP and MOBP with *p* values of 3.40e‐6 and 4.37e‐6, respectively. Only the MAL model remained significant following the Bonferroni correction. All the significant regression models show a positive correlation between gene expression and QSM, with slopes varying from .55 to .76. The subject‐level regression results are also reported for each model in Figure S1g–s, and these show that regression models for CNP, OLIG2, MAL, MOBP, MOG, CLDN11, PLP1, GAL3ST1, PLLP, ILK, OMG, KLK6, and MAG are similar across subjects. The slopes for all of these range from .10 to .77 and the *p* values range from 8.56e‐6 to 5.91e‐1.

### 3.3 Validation with second QSM dataset, two reconstruction methods

**FIGURE 6:**
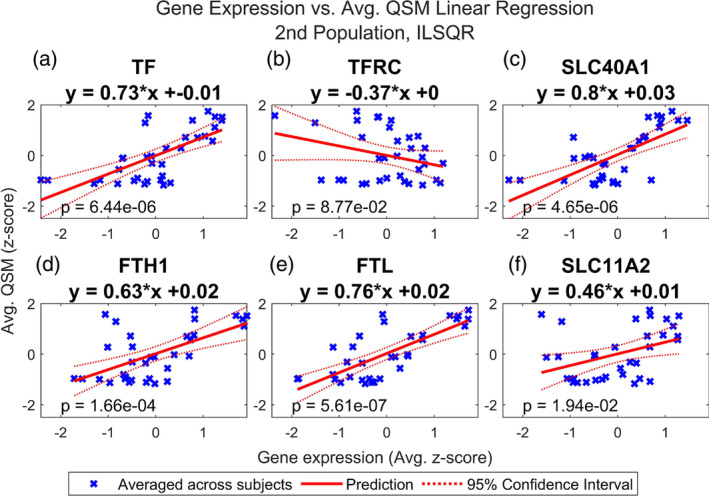
Multiple regression of QSM vs. iron related genes, 2nd population reconstructed with iLSQR. Linear regression of QSM from second population vs. normalized expression of (a) TF, (b) TFRC, (c) SLC40A1, (d) FTH1, (e) FTL, and (f) SLC11A2 in the deep grey nuclei regions. These refer to transferrin (TF), transferrin receptor (TFRC), ferroportin (SLC40A1), ferritin heavy chain (FTH1), ferritin light chain (FTL), and divalent metal transporter 1 (SLC11A2). QSM images from a second set of healthy subjects were reconstructed using iLSQR. QSM and gene expression were averaged across subjects. Regions of interest in the deep grey nuclei are listed in Table 1. See Figure S2 for the results of linear regression with the iron gene set performed for each subject separately.

**FIGURE 7:**
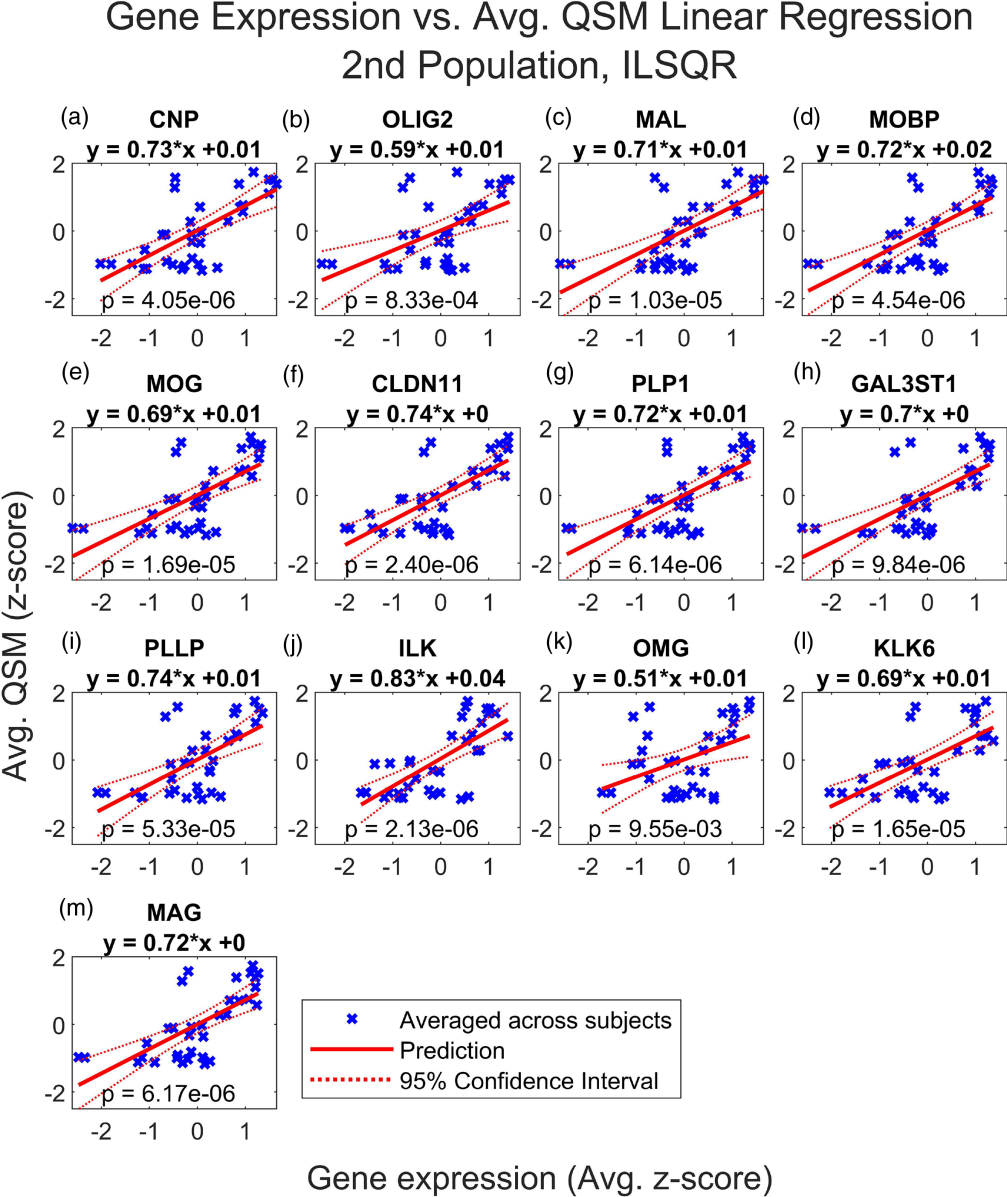
Multiple regression of QSM vs. myelin related genes, 2nd population reconstructed with iLSQR. Linear regression of QSM from second population vs. normalized expression of (a) CNP, (b) OLIG2, (c) MAL, (d) MOBP, (e) MOG, (f) CLDN11, (g) PLP1, (h) GAL3ST1, (i) PLLP, (j) ILK, (k) OMG, (l) KLK6 and (m) MAG in the deep grey nuclei regions. QSM images from a second set of healthy subjects were reconstructed using iLSQR. QSM and gene expression were averaged across subjects. Regions of interest in the deep grey nuclei are listed in Table 1. Only significant results are shown. These include 2′,3′‐cyclic nucleotide 3′‐phosphodiesterase (CNP), oligodendrocyte transcription factor 2 (OLIG2), myelin and lymphocyte protein (MAL), myelin‐associated oligodendrocytic basic protein (MOBP), myelin oligodendrocyte glycoprotein (MOG), claudin‐11 (CLDN11), proteolipid protein (PLP1), galactose‐3‐O‐sulfotransferase‐1 (GAL3ST1), proteolipid plasmolipin (PLLP), integrin‐linked kinase (ILK), oligodendrocyte‐myelin glycoprotein (OMG), kallikrein‐related peptidase 6 (KLK6) and myelin‐associated glycoprotein (MAG). See Figure S2 for the results of linear regression with the myelin gene set performed for each subject separately.

**FIGURE 8:**
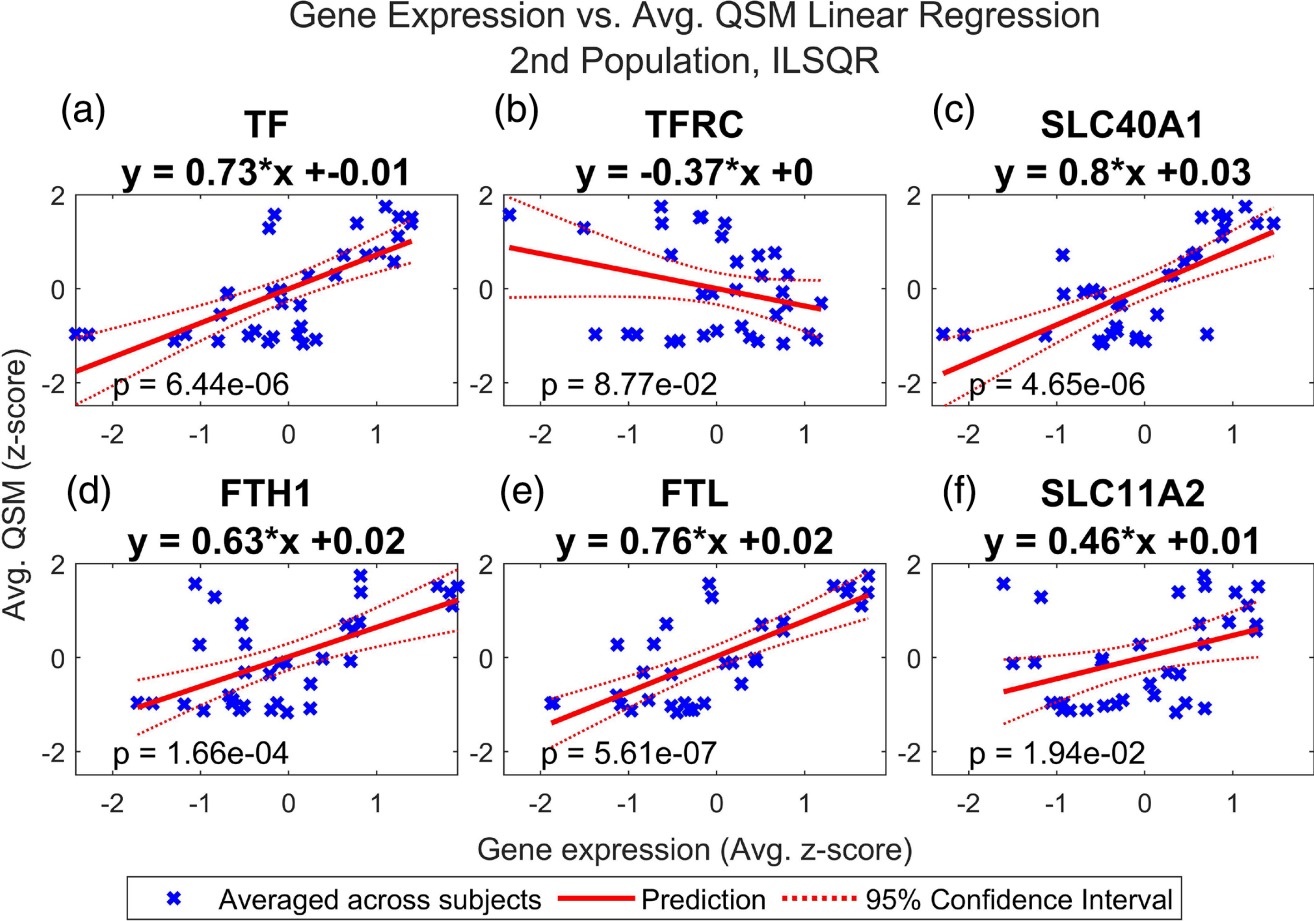
Multiple regression of STAR‐QSM vs. iron related genes, 2nd population reconstructed with STAR‐QSM. Linear regression of QSM from second population vs. normalized expression of (a) TF, (b) TFRC, (c) SLC40A1, (d) FTH1, (e) FTL, and (f) SLC11A2 in the deep grey nuclei regions. These refer to transferrin (TF), transferrin receptor (TFRC), ferroportin (SLC40A1), ferritin heavy chain (FTH1), ferritin light chain (FTL), and divalent metal transporter 1 (SLC11A2). QSM images from a second set of healthy subjects were reconstructed using STAR‐QSM. QSM and gene expression were averaged across subjects. Regions of interest in the deep grey nuclei are listed in Table 1. See Figure S3 for the results of linear regression with the iron gene set performed for each subject separately.

**FIGURE 9:**
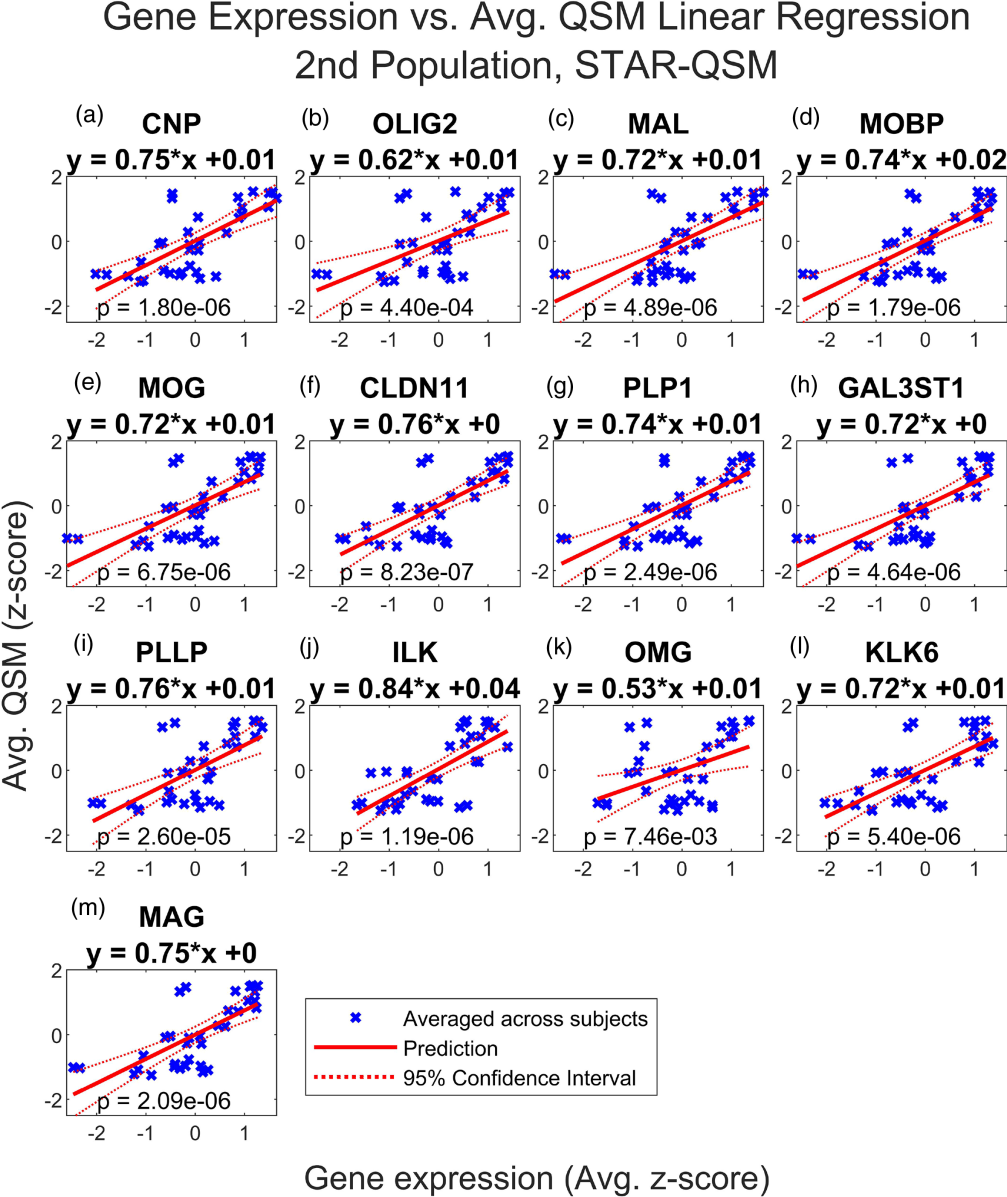
Multiple regression of QSM vs. myelin related genes, 2nd population reconstructed with STAR‐QSM. Linear regression of QSM from second population vs. normalized expression of (a) CNP, (b) OLIG2, (c) MAL, (d) MOBP, (e) MOG, (f) CLDN11, (g) PLP1, (h) GAL3ST1, (i) PLLP, (j) ILK, (k) OMG, (l) KLK6 and (m) MAG in the deep grey nuclei regions. QSM images from a second set of healthy subjects were reconstructed using STAR‐QSM. QSM and gene expression were averaged across subjects. Regions of interest in the deep grey nuclei are listed in Table 1. Only significant results are shown. These include 2′,3′‐cyclic nucleotide 3′‐phosphodiesterase (CNP), oligodendrocyte transcription factor 2 (OLIG2), myelin and lymphocyte protein (MAL), myelin‐associated oligodendrocytic basic protein (MOBP), myelin oligodendrocyte glycoprotein (MOG), claudin‐11 (CLDN11), proteolipid protein (PLP1), galactose‐3‐O‐sulfotransferase‐1 (GAL3ST1), proteolipid plasmolipin (PLLP), integrin‐linked kinase (ILK), oligodendrocyte‐myelin glycoprotein (OMG), kallikrein‐related peptidase 6 (KLK6) and myelin‐associated glycoprotein (MAG). See Figure S3 for the results of linear regression with the myelin gene set performed for each subject separately.

Finally, we repeated this analysis using the average QSM values from the second dataset, reconstructed using both iLSQR and STAR‐QSM. Figures 6a–f, 7a–m, 8a–f, and 9a–m show that the results are comparable to those calculated using the first dataset (reconstructed with STAR‐QSM). As for the first dataset, of the 23 iron and myelination genes considered, we found TF, SLC40A1, FTH1, FTL, and SLC11A2 from the iron set and CNP, OLIG2, MAL, MOBP, MOG, CLDN11, PLP1, GAL3ST1, PLLP, ILK, OMG, KLK6, and MAG from the myelin set to be significant following the Benjamini–Hochberg procedure. We confirmed this result using both the iLSQR and STAR‐QSM reconstruction methods. We again found that the slopes are positive for all the significant regression models, and they are similarly valued across the three QSM datasets. For iLSQR, the slopes range from .46 to .80 for the iron set and from .51 to .83 for the myelin set. For STAR‐QSM, these range from .49 to .80 for the iron set and from .53 to .84 for the myelin set.

Most interestingly, we found FTL to have the smallest *p* value of all the iron genes across the three sets of QSM, with a value of 5.61e‐7 for the set reconstructed with iLSQR and 8.99e‐7 for the set reconstructed with STAR‐QSM. Additionally, the FTL model was the only one out of all genes considered (both iron and myelin) to remain significant following the Bonferroni correction for each of the three sets of QSM. For iLQSR, FTL was the only significant iron gene following the Bonferroni correction, however, TF was also significant following the Bonferroni correction for STAR‐QSM. There is more variation in the most significant myelin regression models among the three QSM datasets, with ILK having the smallest p value at 2.13e‐6 for the set reconstructed with iLSQR and CLDN11 having the smallest p value at 8.23e‐7 for the set reconstructed with STAR‐QSM. This is reflected in the myelin models that are significant following the Bonferroni correction for each reconstruction method, which include CNP, ILK, MAG, MOBP, CLDN11, and PLP1 for STAR‐QSM and ILK and CLDN11 for iLSQR.

The subject‐level regression results are again reported for each model in Figure S2a–s (for iLSQR and 3a‐s for STAR‐QSM), and these show good agreement for the significant regression models. For iLSQR, the slopes of the significant iron regression models range from .24 to .79 and the *p* values range from 1.11e‐5 to 2.15e‐01. The slopes of the significant myelin regression models range from .16 to .82 and the *p* values from 6.49e‐6 to 3.61e‐1. The results are similar for the STAR‐QSM reconstruction method. The slopes of the significant iron set again range from .24 to .79, however, the minimum and maximum *p* values are slightly different at 5.10e‐6 and 2.05e‐1. Similarly, the minimum and maximum slopes of the significant myelin set are the same as for iLSQR, but the *p* values are slightly different, ranging from 2.32e‐6 to 3.05e‐1.

## 4 DISCUSSION

Our analysis revealed a spatial congruent relationship between tissue magnetic susceptibility and gene expression in the brain. We found significant positive correlations between expression of genes encoding both light and heavy chain Ft and several iron transporters, and the QSM signal across these regions. Interestingly, we also observed a positive correlation between QSM and several genes involved in myelination, expressed by oligodendrocytes. This reflects that the iron‐loaded Ft is likely localized to mature oligodendrocytes. Indeed, oligodendrocytes have been found to outnumber neurons in regions of high iron, like the basal ganglia, and iron is localized mainly to oligodendrocytes and the neuropil within these regions (Hill & Switzer, 1984; Valério‐Gomes et al., 2018). In addition, oligodendrocytes are known to require iron in large amounts for myelination (Cheli et al., 2020; Connor & Menzies, 1996). This iron can be acquired by oligodendrocytes via the H‐ferritin receptor (Todorich et al., 2009, 2011).

### 4.1 Iron and myelin related genes are strongly correlated with QSM

We have confirmed the strong relationship between the QSM signal and Ft. Interestingly, our results imply unequal contributions from H‐Ft and L‐Ft, as L‐Ft was found to have the most significant relationship with QSM, among the iron genes, across the three QSM datasets. These are known to exhibit different roles in iron homeostasis and to be differentially distributed across cell types and brain regions, with H‐Ft largely out‐numbering L‐Ft (Connor & Menzies, 1995). In addition to serving as an iron transporter, H‐Ft has the ferroxidase ability, which converts the reactive ferrous iron to more stable ferric iron (Arosio & Levi, 2002). L‐Ft is more important for the formation of the iron core, which is what generates the molecule's strong paramagnetic susceptibility signal (Arosio & Levi, 2002; Levi et al., 1992). Additionally, L‐Ft is present in oligodendrocytes, supporting the hypothesis of their role in iron storage (Connor et al., 1994). This is consistent with the results of our linear regression analysis, which predicts the L‐Ft regression model to be more significant than H‐Ft, regardless of QSM population and reconstruction method.

We know that the major determinant of the QSM signal is magnetic susceptibility. Although several of the myelination genes show a positive correlation with QSM in the deep grey nuclei, which we know to have positive susceptibility, the proteins encoded by these genes actually make up the myelin sheath, which itself has a negative susceptibility. It is possible these genes may be expressed by oligodendrocytes, either containing iron‐loaded Ft or present in the same voxel as other iron‐loaded cells. If the second case, there is a strong likelihood that oligodendrocytes are involved in the transport of iron either to or from these other iron‐loaded cells. The correlation with QSM is gene expression, which reflects oligodendrocyte activity, not necessarily protein content.

### 4.2 Iron transporters are indicated by QSM

The positive correlation of QSM with the expression of Fpn, the only known iron exporter, suggests that the QSM signal reflects not only iron storage, but active transport. More specifically, this result indicates that Fpn must be expressed on cells loaded with iron, or at least, on cells co‐localizing with iron‐loaded cells within voxels of the QSM image. Oligodendrocytes, neurons, microglia, and astrocytes all express Fpn, however, oligodendrocytes accumulate iron in significantly larger amounts than any other cell (Reinert et al., 2019; Wu et al., 2004). Microglia and astrocytes are also known to express Ft, particularly the light chain subunit which is associated with iron storage, and there is evidence that both cells may release iron loaded H‐Ft for use by oligodendrocytes early in development (Todorich et al., 2009; Zhang et al., 2006). Astrocytes are also implicated in providing growth factors necessary for maturation of oligodendrocyte precursor cells (Schulz et al., 2012; Suh & David, 2006). Although the density of microglia is particularly high in the basal ganglia, even accounting for 12% of cells in the substantia nigra of the adult mouse brain (Lawson et al., 1990), microglia show significant variation in their transcriptome by brain region, particularly among the deep grey nuclei (de Biase et al., 2017; Grabert et al., 2016). Therefore, it seems unlikely that the positive correlation between gene expression of iron transporters and QSM across deep grey nuclei could be due to these cells. In fact, microglial cell number in the deep grey nuclei is not correlated with oligodendrocyte density, but rather, that of astrocytes (de Biase et al., 2017). Astrocytes, despite expressing TFR, DMT1, Fpn, and Ft, maintain a low level of cellular iron (Cheli et al., 2020). This evidence, taken together, again points to oligodendrocytes as the source of the QSM signal.

The positive correlation of Tf with QSM can also be explained by the presence of oligodendrocytes. Tf is a well‐known marker of oligodendrocytes, as it is secreted by them and most Tf in the brain is thought to originate from these cells (Bloch et al., 1985; Dwork et al., 1988; Morris et al., 1992). Despite this, mature oligodendrocytes don't express TFR, instead likely getting most of their iron via H‐Ft (Hill et al., 1985). In this context, the lack of correlation between TFR expression and the QSM signal makes sense and is consistent with the known distribution of TFR in the brain (Hill et al., 1985). Although TFR is expressed in central neurons, the distribution, found from histology studies, doesn't reflect areas of high iron concentration (Bradbury, 1997; Hill et al., 1985). This supports the theory of H‐Ft as the major route of iron transport for mature oligodendrocytes (Todorich et al., 2011). Indeed, receptors for H‐Ft, specifically T‐cell immunoglobulin and mucin domain (Tim‐1), exist on myelinating oligodendrocytes (Chiou et al., 2018), and these cells are known to contain the vast majority of iron in the brain (Connor & Menzies, 1995). This is likely because iron is required in high concentrations for the synthesis of myelin (Cheli et al., 2020; Connor & Menzies, 1996). Additionally, it has been shown that neurons in regions of high TFR expression project to regions with high iron (Hill et al., 1985; Moos & Morgan, 1998). Because of this, (Wang et al., 2019) have explored the possibility of axonal transport of iron between such regions and have identified two different pathways. The results of our study support the idea that the role of TFR is mainly one of iron uptake from the blood brain barrier, and that further iron transport between regions is responsible for iron accumulation in various deep grey nuclei.

With this information, it seems likely that TFR expression mainly reflects influx of iron across the BBB rather than iron transport between brain regions. This is supported by evidence showing that iron in the developing brain is first seen in oligodendrocyte progenitor cells near blood vessels, only later moving to sites of myelination (Todorich et al., 2009). DMT1 is another potential route of iron transport, found in both the glia and neurons, however it is not specific to iron (Skjørringe et al., 2015). Despite this, we found a significant positive correlation (FDR 5%) between DMT1 gene expression and QSM in the deep grey nuclei. Unlike TFR, DMT1 has been found to be present in higher amounts in the striatum than in the cortex in the rat brain, however, throughout the entire brain it is localized mainly to BMECs and ependymal cells (Burdo et al., 2001; Skjørringe et al., 2015). Within BMECs, DMT1 and TFR have been shown to co‐localize, and DMT1 has also been found on the end‐feet of astrocytes, which interface with BMECs (Burdo et al., 2001). This points to DMT1 being involved in the process of iron uptake, along with TFR. The presence of DMT1 in ependymal cells may indicate it plays a role in iron exchange between the brain interstitial fluid and the ventricles (Burdo et al., 2001). Interestingly, (Cheli et al., 2018, 2023) have shown that DMT1 also co‐localizes with TFR in oligodendrocyte progenitor cells, and that both of these are upregulated during the beginning stages of oligodendrocyte progenitor cell maturation. Additionally, DMT1 and TFR appear to be required for the iron accumulation and morphological development of these cells, both of which are precursors to myelination (Cheli et al., 2018, 2023). However, there is evidence that DMT1 is necessary for normal myelination even in the adult brain, while TFR may not influence mature oligodendrocytes or myelination (Cheli et al., 2018, 2023). Therefore, it is possible that high expression of TFR is more reflective of oligodendrocyte progenitor cells, rather than mature, myelinating oligodendrocytes, which seem to be more relevant to the QSM signal, as discussed next.

### 4.3 QSM may reflect presence of mature oligodendrocytes

The myelination genes found to be significantly positively correlated with QSM at 5% FDR are markers of mature oligodendrocytes (Chavarria‐Siles et al., 2015). These include CNP, MAG, MAL, MOBP, MOG, CLDN11, PLP1, GAL3ST1, PLLP, ILK, OMG, KLK6, and OLIG2. Oligodendrocytes are mitotic cells, starting as simple, migratory cells then moving to various regions of the brain and changing to become morphologically more complex (Baumann & Pham‐Dinh, 2001; Kuhn et al., 2019). This involves extending processes from the soma, down‐regulating TFR, and accumulating iron in large amounts, potentially via the H‐Ft receptor (Todorich et al., 2011). Various genes are expressed sequentially by oligodendrocytes to facilitate this process of development (Kuhn et al., 2019). CNP encodes a protein that, among other functions, is implicated in the formation of process outgrowths in oligodendrocyte progenitor cells (Fulton et al., 2010). It is also a major constituent of the myelin sheath, along with myelin basic protein (MBP) and PLP1 (Fulton et al., 2010). These proteins make up the largest structural contribution to the myelin sheath, and are expressed early on in myelination, along with ILK and OLIG2 which are required for oligodendrocyte maturation (Chun et al., 2003; Frank et al., 1999; Mei et al., 2013). MAG is also expressed early in the process of myelination and continues to be expressed following oligodendrocyte maturation, indicating a potential role in myelin maintenance (Quarles, 2007). KLK6 may be involved in regulating oligodendrocyte differentiation (Yoon et al., 2022). PLLP is implicated in myelin synthesis, as well as remyelination, and has been found to co‐localize with MAG (Shulgin et al., 2021; Yaffe et al., 2015).

MAL, MOBP, MOG, and OMG are expressed later, and have various roles important for the compaction and stabilization of myelin (Frank et al., 1999; Holz & Schwab, 1997; Montague et al., 2006; Vourc'h et al., 2003). MAL is one of the last genes to be expressed in myelination and is located within the compact myelin (Frank et al., 1999; Schaeren‐Wiemers et al., 2004). CLDN11 is essential for the formation of tight junctions between layers of the myelin sheath, which are necessary for the fast transmission of electrical signals down the axon (Denninger et al., 2015; Maheras et al., 2018). GAL3ST1 encodes an enzyme required for the maintenance of proper myelin sheath structure (Marcus et al., 2006; Ramakrishnan et al., 2007). All together, these genes typically reflect the presence of myelinating oligodendrocytes, however, they are also known to be expressed, but not translated, in nonmyelinating perineuronal oligodendrocytes (Baumann & Pham‐Dinh, 2001). These are implicated in remyelination and may be involved in mediating iron transport or other trophic factors for use by neurons, as they have much higher metabolic requirements than neurons despite not producing myelin (Du & Dreyfus, 2002; Gerber & Connor, 1989). Biologically it is unclear why MAL, ILK, and CLDN11 are more significant than the other myelination genes in our statistical analyses. Nonetheless, the result is consistent with the fact that each has the smallest *p* value among all myelination genes as found in the multiple regression analyses with three QSM datasets.

### 4.4 The relationship between QSM and gene expression is consistent across subject pool and reconstruction method

Figures 1 and 2 show that the average QSM signal calculated across deep grey nuclei regions is comparable between the two datasets when using the same reconstruction algorithm, although the susceptibility values calculated from images reconstructed with STAR‐QSM are generally smaller compared to those calculated from images reconstructed with iLSQR. The average susceptibility values across deep grey nuclei regions show good agreement to those reported in the studies describing the collection of both datasets (Cogswell et al., 2021; Zhang et al., 2018). Slight differences in susceptibility found in the same region may be due to registration errors or subject variability, a consequence of using a small sample size for each dataset. Despite the discrepancies in susceptibility between the two datasets, we achieve remarkably similar results in our regression analysis. These results hold when using the susceptibility values calculated from images reconstructed with iLSQR as well.

We also compare our calculated average susceptibility values to those measured using multi‐orientation phase acquisition on a 7T scanner and calculation of susceptibility through multiple orientation sampling (COSMOS), reported in (Deistung et al., 2013). There is some difference between our values and those obtained using COSMOS (Figure S7). This discrepancy is mostly due to the use of the frontal deep white matter as a reference in (Deistung et al., 2013), whereas our reported susceptibility values were obtained using the whole brain mean as a reference. White matter has diamagnetic susceptibility, which, when subtracted, will increase the susceptibility of the basal ganglia regions. Our results are comparable to values reported in (Cogswell et al., 2021) and (Zhang et al., 2018), suggesting that registration error is not overly significant. It is possible that some discrepancies in the susceptibility values may be due to the choice of atlas, however, (Zhang et al., 2018) compared the results of susceptibility values in the deep grey nuclei calculated using the age‐specific QSM atlas to those calculated using a manual segmentation drawn by three radiologists and found that using the age‐specific QSM atlas yielded susceptibility values comparable to those determined from the ROIs drawn by radiologists. In order to investigate this further, we repeated our regression analysis using the values reported in (Deistung et al., 2013), which were averaged across the hemispheres, and obtained similar results except for TFRC, reported in Figures [Link], [Link]. The (Deistung et al., 2013) dataset yielded a regression model for TFRC with a much larger negative slope, and this model was found to be significant following the Benjamini–Hochberg procedure. This discrepancy may be a result of the QSM acquisition methodology used by (Deistung et al., 2013), however, it is an interesting finding as the negative correlation between TFRC and QSM supports the theory of iron accumulation in the deep grey nuclei being driven by mature oligodendrocytes (Cheli et al., 2018, 2023). Although TFR is used to import iron across the BBB, it is downregulated in mature oligodendrocytes (Todorich et al., 2011). This supplementary analysis, although limited by fewer QSM datapoints, strengthens the results of our original findings.

## 5 LIMITATIONS

Our analysis is limited by a small subject pool, and the reliance upon two separate subject pools for the QSM and gene expression datasets. However, in the brain, many genes are known to exhibit differential expression throughout regions (Hawrylycz et al., 2012), and this specific expression pattern has been found to be highly conserved among individuals, with the vast majority of genes reflecting consistent spatially determined patterns of expression between individual brains (Zeng et al., 2012). This is explored further in Figure S6. Differences between subjects, particularly age and post‐mortem tissue collection in the formation of the AHBA dataset, may explain some discrepancies in the gene expression data. Only two out of the six subjects had regions sampled from both hemispheres of the brain, rather than just the left hemisphere. Notably, myelin basic protein (MBP) was not found to be significantly correlated with QSM. MBP is usually expressed by mature oligodendrocytes, around the time as other genes like CNP and PLP1, and it is typically used as a marker of oligodendrocytes reaching the last developmental stage (Frank et al., 1999; Fulton et al., 2010). The reason for MBP's absence is unclear, potentially it is a result of differences in tissue collection between subjects, or it may be a result of the probe chosen to measure MBP in the AHBA dataset. MBP is known to be a long gene with multiple transcripts, each encoding a different protein (Fulton et al., 2010).

Our method of image acquisition also comes with certain limitations and trade‐offs. In particular, the susceptibility values we determined from ROI analysis were obtained using single‐orientation phase data. COSMOS is considered to be more reliable as the collection of multiple phase images at different orientations stabilizes the dipole inversion problem, resulting in QSM images less susceptible to streaking artifacts and susceptibility underestimation (Liu et al., 2009). This method, although more accurate, requires a significantly longer scan time, making data acquisition far less practical than reconstruction from a single phase image. Our use of the STAR‐QSM and iLSQR algorithms for reconstruction of a single‐orientation 3D phase image may have resulted in slightly different QSM values (Figure S7). In order to explore this more, we repeated our regression analysis using the susceptibility values reported in (Deistung et al., 2013). Figures [Link], [Link] show that these results are comparable to those reported in Figures 4, 5, 6, 7, 8, 9, supporting the validity of our original results. This analysis, however, is limited by the small number of ROIs reported by (Deistung et al., 2013), which are averaged over both hemispheres. It would be interesting to apply our analytical methods to a larger QSM dataset, acquired using COSMOS.

Additionally, the use of mRNA as a proxy for protein concentration comes with limitations. Various proteins, including Ft, are known to be post‐transcriptionally regulated, meaning mRNA may not be representative of protein concentration (Han et al., 2002). Despite this, our results clearly show a correlation between the QSM signal, which has been found to be linearly related to Ft protein concentration in the deep grey nuclei, and Ft gene expression. Errors may also be introduced in the QSM registration process. Finally, we are limited by the set of genes included in the AHBA dataset. For instance, Tim‐1 appears to be relevant to iron homeostasis, yet it is not present in the AHBA microarray survey (Chiou et al., 2018).

We restricted our analysis to the deep grey nuclei because that is where the QSM signal most accurately reflects iron concentration. In other regions of the brain where iron is also stored but in smaller amounts, such as the cortex, the positive susceptibility of iron within a voxel may be cancelled out by negative susceptibilities of other molecules, like myelin. In future, using algorithms designed to separate out the sub‐voxel positive and negative susceptibility contributions, like DECOMPOSE‐QSM (Chen et al., 2021), would allow for a more accurate measurement of iron in the brain, and would allow us to extend our analysis to regions outside the deep grey nuclei. Even in the deep grey nuclei, the positive susceptibility of iron dominates over other species in QSM. It would be interesting to redo our analyses with paramagnetic and diamagnetic susceptibility maps, especially in light of our finding that the correlations between QSM and multiple myelin genes, including MAL, ILK, and CLDN11, are highly significant. MAL and CLDN11 are known to be present in compact myelin which is diamagnetic, however, we are unable to measure the contributions of diamagnetic species in the deep grey nuclei with QSM alone.

## 6 CONCLUSIONS

Our results show a positive correlation between QSM and expression of genes important for iron transport and storage and myelination across regions of the deep grey nuclei. This seemingly contradictory result likely points to the presence of oligodendrocytes in voxels containing iron‐loaded Ft. Our analysis verifies the work of previous studies showing a relationship between QSM and genes relevant to iron homeostasis and myelination in the deep grey nuclei and expands upon these studies by using spatially localized gene expression data from the AHBA (Benyamin et al., 2014; Elliott et al., 2018; Wang et al., 2022). In addition, we have demonstrated the robustness of our result by repeating our analysis with two different QSM populations and two different QSM reconstruction methods, iLSQR and STAR‐QSM. It is clear QSM is an informative measure of iron homeostasis and shows valuable promise for use in understanding the complex pattern of iron accumulation in the brain.

## FUNDING INFORMATION

The study is supported in part by the National Institutes of Health (NIH) through grants R01AG070826, R01MH127104, and U01AG06786, and by the Alzheimer's Drug Discovery Foundation through grant GC‐201810‐2017383.

## CONFLICT OF INTEREST STATEMENT

The authors declare no conflicts of interest.

## Supporting information


**FIGURE S1.** Subject‐level multiple regression of QSM vs. iron and myelin related genes. Linear regression of QSM vs. normalized expression of (a) TF, (b) TFRC, (c) SLC11A2, (d) SLC40A1, (e) FTH1, (f) FTL, (g) CNP, (h) OLIG2, (i) MAL, (j) MOBP, (k) MOG, (l) CLDN11, (m) PLP1, (n) GAL3ST1, (o) PLLP, (p) ILK, (q) OMG, (r) KLK6 and (s) MAG in the deep grey nuclei regions. These refer to transferrin (TF), transferrin receptor (TFRC), divalent metal transporter 1 (SLC11A2), ferroportin (SLC40A1), ferritin heavy chain (FTH1), ferritin light chain (FTL), 2′,3′‐cyclic nucleotide 3′‐phosphodiesterase (CNP), oligodendrocyte transcription factor 2 (OLIG2), myelin and lymphocyte protein (MAL), myelin‐associated oligodendrocytic basic protein (MOBP), myelin oligodendrocyte glycoprotein (MOG), claudin‐11 (CLDN11), proteolipid protein (PLP1), galactose‐3‐O‐sulfotransferase‐1 (GAL3ST1), proteolipid plasmolipin (PLLP), integrin‐linked kinase (ILK), oligodendrocyte‐myelin glycoprotein (OMG), kallikrein‐related peptidase 6 (KLK6) and myelin‐associated glycoprotein (MAG). Regressions were performed for each subject in the Allen Human Brain Atlas (AHBA). QSM was averaged across subjects and across regions. Regions of interest in the deep grey nuclei are listed in Table 1.

**FIGURE S2.** Subject‐level multiple regression of QSM vs. iron and myelin related genes, 2nd population reconstructed with iLSQR. Linear regression of QSM vs. normalized expression of (a) TF, (b) TFRC, (c) SLC11A2, (d) SLC40A1, (e) FTH1, (f) FTL, (g) CNP, (h) OLIG2, (i) MAL, (j) MOBP, (k) MOG, (l) CLDN11, (m) PLP1, (n) GAL3ST1, (o) PLLP, (p) ILK, (q) OMG, (r) KLK6 and (s) MAG in the deep grey nuclei regions. These refer to transferrin (TF), transferrin receptor (TFRC), divalent metal transporter 1 (SLC11A2), ferroportin (SLC40A1), ferritin heavy chain (FTH1), ferritin light chain (FTL), 2′,3′‐cyclic nucleotide 3′‐phosphodiesterase (CNP), oligodendrocyte transcription factor 2 (OLIG2), myelin and lymphocyte protein (MAL), myelin‐associated oligodendrocytic basic protein (MOBP), myelin oligodendrocyte glycoprotein (MOG), claudin‐11 (CLDN11), proteolipid protein (PLP1), galactose‐3‐O‐sulfotransferase‐1 (GAL3ST1), proteolipid plasmolipin (PLLP), integrin‐linked kinase (ILK), oligodendrocyte‐myelin glycoprotein (OMG), kallikrein‐related peptidase 6 (KLK6) and myelin‐associated glycoprotein (MAG). Regressions were performed for each subject in the Allen Human Brain Atlas (AHBA). QSM was averaged across subjects and across regions. Regions of interest in the deep grey nuclei are listed in Table 1.

**FIGURE S3.** Subject‐level multiple regression of QSM vs. iron and myelin related genes, 2nd population reconstructed with STAR‐QSM. Linear regression of QSM vs. normalized expression of (a) TF, (b) TFRC, (c) SLC11A2, (d) SLC40A1, (e) FTH1, (f) FTL, (g) CNP, (h) OLIG2, (i) MAL, (j) MOBP, (k) MOG, (l) CLDN11, (m) PLP1, (n) GAL3ST1, (o) PLLP, (p) ILK, (q) OMG, (r) KLK6 and (s) MAG in the deep grey nuclei regions. These refer to transferrin (TF), transferrin receptor (TFRC), divalent metal transporter 1 (SLC11A2), ferroportin (SLC40A1), ferritin heavy chain (FTH1), ferritin light chain (FTL), 2′,3′‐cyclic nucleotide 3′‐phosphodiesterase (CNP), oligodendrocyte transcription factor 2 (OLIG2), myelin and lymphocyte protein (MAL), myelin‐associated oligodendrocytic basic protein (MOBP), myelin oligodendrocyte glycoprotein (MOG), claudin‐11 (CLDN11), proteolipid protein (PLP1), galactose‐3‐O‐sulfotransferase‐1 (GAL3ST1), proteolipid plasmolipin (PLLP), integrin‐linked kinase (ILK), oligodendrocyte‐myelin glycoprotein (OMG), kallikrein‐related peptidase 6 (KLK6) and myelin‐associated glycoprotein (MAG). Regressions were performed for each subject in the Allen Human Brain Atlas (AHBA). QSM was averaged across subjects and across regions. Regions of interest in the deep grey nuclei are listed in Table 1.

**FIGURE S4.** Correlation between expression of iron and myelin related genes across deep grey nuclei. The genes listed in column 1 refer to the full set of iron and myelin genes. Gene expression vectors are the normalized expression of a given gene across deep grey nuclei regions (listed in Table 1), averaged over all Allen Human Brain Atlas (AHBA) subjects. (a) Correlation coefficients across iron gene expression vectors. (b) Correlation coefficients across myelin gene expression vectors (significant only).

**FIGURE S5.** Matching QSM and Allen Human Brain Atlas (AHBA) segmentations. In some instances, like the hippocampus, we combined multiple AHBA samples if they fell within the same region, as defined in the QSM segmentation. (a) QSM with hippocampus region segmented. (b) AHBA subdivision of hippocampus (Allen Reference Atlas—Adult Human, human.brain-map.org and atlas.brain-map.org; Ding et al., 2016). Note that the dentate gyrus (DG), CA1 field, CA2 field, CA3 field, CA4 field, and subiculum (Sub) are sampled in the AHBA segmentation, however, we only calculated the average QSM across the entire hippocampus.

**FIGURE S6.** Bootstrapping QSM and gene expression linear regression. Resampling three out of the nine QSM subjects, 1st Population, and using the average of only these subjects to perform linear regression with (a) TF gene expression and (b) CNP gene expression. Both results yield distributions with modes that are in close range to the regression coefficient reported using all QSM subjects averaged.

**FIGURE S7.** Average QSM in Deep Grey Matter Regions, Deistung et al., 2013. (a) Average QSM (b) Normalized average QSM. Regions of interest on the x‐axis correspond to Putamen (Pu), Caudate Nucleus (CN), External Globus Pallidus (ExtGP), Internal Globus Pallidus (IntGP), Substantia Nigra pars reticulata (SNpr), Substantia Nigra pars compacta (SNpc), Red Nucleus (RN), Subthalamic nuclei (SubTl), Anterior nuclei of the Thalamus (AntTl), Median nuclei of the Thalamus (MedTl), Lateral nuclei of the Thalamus (LatTl), and Pulvinar nuclei of the Thalamus (PulTl). These correspond to the mean susceptibility values reported in (Deistung et al., 2013), which are averaged across both hemispheres. The susceptibility values of SNpr and SNpc are the same because these regions aren't distinguished for the value reported for SN in (Deistung et al., 2013).

**FIGURE S8.** Multiple regression of QSM vs. iron related genes, Deistung et al., 2013. Linear regression of QSM from (Deistung et al., 2013) vs. normalized expression of (a) TF, (b) TFRC, (c) SLC40A1, (d) FTH1, (e) FTL, and (f) SLC11A2 in deep grey nuclei regions. These refer to transferrin (TF), transferrin receptor (TFRC), ferroportin (SLC40A1), ferritin heavy chain (FTH1), ferritin light chain (FTL), and divalent metal transporter 1 (SLC11A2). QSM and gene expression were averaged across subjects. Regions of interest in the deep grey nuclei are listed in Figure S7. See Figure S10 for the results of linear regression with the iron gene set performed for each subject separately.

**FIGURE S9.** Multiple regression of QSM vs. myelin related genes, Deistung et al., 2013. Linear regression of QSM from (Deistung et al., 2013) vs. normalized expression of (a) CNP, (b) ILK, (c) MAG, (d) MAL, (e) MBP, (f) MOBP, (g) MOG, (h) CLDN11, (i) PLP1, (j) KLK6, (k) GAL3ST1, and (l) PLLP in deep grey nuclei regions. QSM and gene expression were averaged across subjects. Regions of interest in the deep grey nuclei are listed in Figure S7. Only significant results are shown. These include 2′,3′‐cyclic nucleotide 3′‐phosphodiesterase (CNP), integrin‐linked kinase (ILK), myelin‐associated glycoprotein (MAG), myelin and lymphocyte protein (MAL), myelin basic protein (MBP), myelin‐associated oligodendrocytic basic protein (MOBP), myelin oligodendrocyte glycoprotein (MOG), claudin‐11 (CLDN11), proteolipid protein (PLP1), kallikrein‐related peptidase 6 (KLK6), galactose‐3‐O‐sulfotransferase‐1 (GAL3ST1), and proteolipid plasmolipin (PLLP). See Figure S10 for the results of linear regression with the myelin gene set performed for each subject separately.

**FIGURE S10.** Subject‐level multiple regression of QSM vs. iron and myelin related genes, Deistung et al., 2013. Linear regression of QSM vs. normalized expression of (a) TF, (b) TFRC, (c) SLC11A2, (d) SLC40A1, (e) FTH1, (f) FTL, (g) CNP, (h) MBP, (i) MAL, (j) MOBP, (k) MOG, (l) CLDN11, (m) PLP1, (n) GAL3ST1, (o) PLLP, (p) ILK, (q) KLK6, and (r) MAG in deep grey nuclei regions. These refer to transferrin (TF), transferrin receptor (TFRC), divalent metal transporter 1 (SLC11A2), ferroportin (SLC40A1), ferritin heavy chain (FTH1), ferritin light chain (FTL), 2′,3′‐cyclic nucleotide 3′‐phosphodiesterase (CNP), myelin basic protein (MBP), myelin and lymphocyte protein (MAL), myelin‐associated oligodendrocytic basic protein (MOBP), myelin oligodendrocyte glycoprotein (MOG), claudin‐11 (CLDN11), proteolipid protein (PLP1), galactose‐3‐O‐sulfotransferase‐1 (GAL3ST1), proteolipid plasmolipin (PLLP), integrin‐linked kinase (ILK), kallikrein‐related peptidase 6 (KLK6) and myelin‐associated glycoprotein (MAG). Regressions were performed for each subject in the Allen Human Brain Atlas (AHBA). QSM was averaged across subjects and across regions. Regions of interest in the deep grey nuclei are listed in Figure S7.

**TABLE S1.** Full set of myelination related genes used in linear regression analysis. The regression results were reported only for myelin genes significantly correlated with QSM, following the Benjamini–Hochburg procedure. The abbreviation for each gene and the corresponding protein encoded by the gene are listed in the table above.

**TABLE S2.** Full set of iron homeostasis genes used in linear regression analysis. The regression results were reported for all of these genes. The abbreviation for each gene and the corresponding protein encoded by the gene are listed in the table above.

## Data Availability

The QSM data that supports the findings of this study are available on request from the corresponding author. The data are not publicly available due to privacy or ethical restrictions. The processed gene expression data and code are available in qsm‐gene‐analysis at https://github.com/LiuCLab/qsm-gene-analysis. These data were derived from the following resources available in the public domain: Allen Human Brain Atlas: Microarray at human.brain-map.org, and https://github.com/BMHLab/AHBAprocessing from (Arnatkevic̆iūtė et al., 2019).

## Footnotes

^1^
Allen Reference Atlas ‐ Adult Human, human.brain-map.org and atlas.brain-map.org
