# Supplementary material for: Quantitative susceptibility mapping in the brain reflects spatial expression of genes involved in iron homeostasis and myelination
Source: Hum Brain Mapp. 2024 Jun 19;45(9):e26688. doi: 10.1002/hbm.26688 (PMC11187871; doi:10.1002/hbm.26688)
Supplement: Supplementary file 3 — FIGURE S3. Subject‐level multiple regression of QSM vs. iron and myelin related genes, 2nd population reconstructed with STAR‐QSM. Linear regression of QSM vs. normalized expression of (a) TF, (b) TFRC, (c) SLC11A2, (d) SLC40A1, (e) FTH1, (f) FTL, (g) CNP, (h) OLIG2, (i) MAL, (j) MOBP, (k) MOG, (l) CLDN11, (m) PLP1, (n) GAL3ST1, (o) PLLP, (p) ILK, (q) OMG, (r) KLK6 and (s) MAG in the deep grey nuclei regions. These refer to transferrin (TF), transferrin receptor (TFRC), divalent metal transporter 1 (SLC11A2), ferroportin (SLC40A1), ferritin heavy chain (FTH1), ferritin light chain (FTL), 2′,3′‐cyclic nucleotide 3′‐phosphodiesterase (CNP), oligodendrocyte transcription factor 2 (OLIG2), myelin and lymphocyte protein (MAL), myelin‐associated oligodendrocytic basic protein (MOBP), myelin oligodendrocyte glycoprotein (MOG), claudin‐11 (CLDN11), proteolipid protein (PLP1), galactose‐3‐O‐sulfotransferase‐1 (GAL3ST1), proteolipid plasmolipin (PLLP), integrin‐linked kinase (ILK), oligodendrocyte‐myelin glycoprotein (OMG), kallikrein‐related peptidase 6 (KLK6) and myelin‐associated glycoprotein (MAG). Regressions were performed for each subject in the Allen Human Brain Atlas (AHBA). QSM was averaged across subjects and across regions. Regions of interest in the deep grey nuclei are listed in Table 1. [file HBM-45-e26688-s001.pdf]

(a) **TF Regression**

Subject	Slope	P-Value
1	0.70	5.10E-06
2	0.58	3.69E-04
3	0.71	1.51E-03
4	0.73	1.30E-03
5	0.74	7.03E-04
6	0.74	7.10E-04

(b) **TFRC Regression**

Subject	Slope	P-Value
1	-0.27	1.31E-01
2	-0.50	2.64E-03
3	0.29	2.55E-01
4	0.41	1.14E-01
5	-0.28	2.75E-01
6	0.01	9.60E-01

(c) **SLC11A2 Regression**

Subject	Slope	P-Value
1	0.32	6.65E-02
2	0.24	1.66E-01
3	0.32	2.05E-01
4	0.58	1.77E-02
5	0.56	1.93E-02
6	0.54	2.43E-02

(d) **SLC40A1 Regression**

Subject	Slope	P-Value
1	0.59	3.42E-04
2	0.53	1.30E-03
3	0.60	1.02E-02
4	0.76	5.69E-04
5	0.53	2.84E-02
6	0.75	5.92E-04

(e) **FTH1 Regression**

Subject	Slope	P-Value
1	0.62	1.09E-04
2	0.40	1.93E-02
3	0.60	1.15E-02
4	0.74	1.12E-03
5	0.69	2.22E-03
6	0.72	1.16E-03

(f) **FTL Regression**

Subject	Slope	P-Value
1	0.68	1.48E-05
2	0.66	2.20E-05
3	0.75	5.19E-04
4	0.78	3.21E-04
5	0.68	2.72E-03
6	0.79	1.60E-04

(g) **CNP Regression**

Subject	Slope	P-Value
1	0.65	4.80E-05
2	0.63	5.85E-05
3	0.68	2.84E-03
4	0.73	1.31E-03
5	0.76	4.18E-04
6	0.77	3.08E-04

(h) **OLIG2 Regression**

Subject	Slope	P-Value
1	0.34	5.09E-02
2	0.48	4.15E-03
3	0.69	2.12E-03
4	0.68	3.60E-03
5	0.68	2.80E-03
6	0.70	1.72E-03

(i) **MAL Regression**

Subject	Slope	P-Value
1	0.66	3.00E-05
2	0.56	5.02E-04
3	0.68	2.46E-03
4	0.75	8.36E-04
5	0.75	4.91E-04
6	0.77	3.43E-04

(j) **MOBP Regression**

Subject	Slope	P-Value
1	0.72	2.32E-06
2	0.63	7.26E-05
3	0.66	3.75E-03
4	0.74	1.05E-03
5	0.71	1.27E-03
6	0.74	6.77E-04

(k) **MOG Regression**

Subject	Slope	P-Value
1	0.66	2.83E-05
2	0.59	2.60E-04
3	0.62	8.36E-03
4	0.71	2.19E-03
5	0.75	5.24E-04
6	0.76	3.90E-04

(l) **CLDN11 Regression**

Subject	Slope	P-Value
1	0.67	2.22E-05
2	0.68	8.16E-06
3	0.67	3.23E-03
4	0.74	9.38E-04
5	0.74	6.48E-04
6	0.75	4.89E-04

(m) **PLP1 Regression**

Subject	Slope	P-Value
1	0.70	5.03E-06
2	0.62	9.91E-05
3	0.70	1.94E-03
4	0.70	2.31E-03
5	0.74	7.08E-04
6	0.74	6.50E-04

(n) **GAL3ST1 Regression**

Subject	Slope	P-Value
1	0.66	2.53E-05
2	0.58	3.26E-04
3	0.57	1.59E-02
4	0.74	1.17E-03
5	0.66	4.14E-03
6	0.72	1.22E-03

(o) **PLLP Regression**

Subject	Slope	P-Value
1	0.56	7.66E-04
2	0.41	1.71E-02
3	0.50	4.07E-02
4	0.67	4.62E-03
5	0.70	1.85E-03
6	0.73	8.30E-04

(p) **ILK Regression**

Subject	Slope	P-Value
1	0.52	1.91E-03
2	0.57	4.42E-04
3	0.74	6.57E-04
4	0.83	6.79E-05
5	0.48	4.88E-02
6	0.73	9.70E-04

(q) **OMG Regression**

Subject	Slope	P-Value
1	0.18	3.05E-01
2	0.35	4.26E-02
3	0.44	7.49E-02
4	0.62	1.08E-02
5	0.45	7.01E-02
6	0.64	5.98E-03

(r) **KLK6 Regression**

Subject	Slope	P-Value
1	0.63	7.39E-05
2	0.64	4.00E-05
3	0.64	5.26E-03
4	0.71	2.01E-03
5	0.71	1.54E-03
6	0.70	1.63E-03

(s) **MAG Regression**

Subject	Slope	P-Value
1	0.67	2.09E-05
2	0.63	7.32E-05
3	0.64	5.58E-03
4	0.73	1.29E-03
5	0.72	1.04E-03
6	0.75	5.53E-04
