# Supplementary material for: Quantitative susceptibility mapping in the brain reflects spatial expression of genes involved in iron homeostasis and myelination
Source: Hum Brain Mapp. 2024 Jun 19;45(9):e26688. doi: 10.1002/hbm.26688 (PMC11187871; doi:10.1002/hbm.26688)
Supplement: Supplementary file 4 — FIGURE S4. Correlation between expression of iron and myelin related genes across deep grey nuclei. The genes listed in column 1 refer to the full set of iron and myelin genes. Gene expression vectors are the normalized expression of a given gene across deep grey nuclei regions (listed in Table 1), averaged over all Allen Human Brain Atlas (AHBA) subjects. (a) Correlation coefficients across iron gene expression vectors. (b) Correlation coefficients across myelin gene expression vectors (significant only). [file HBM-45-e26688-s012.pdf]

(a)

Correlation Coefficients across Iron Gene Expression Vectors

	TF	TFRC	SLC40A1	FTH1	FTL	SLC11A2
TF	1.00	0.29	0.78	0.77	0.72	0.81
TFRC	0.29	1.00	0.05	0.29	0.00	0.53
SLC40A1	0.78	0.05	1.00	0.65	0.75	0.52
FTH1	0.77	0.29	0.65	1.00	0.92	0.69
FTL	0.72	0.00	0.75	0.92	1.00	0.51
SLC11A2	0.81	0.53	0.52	0.69	0.51	1.00
CNP	0.97	0.29	0.75	0.83	0.76	0.82
ILK	0.85	0.18	0.77	0.60	0.56	0.75
MAG	0.99	0.29	0.77	0.75	0.71	0.79
MAL	0.96	0.29	0.77	0.89	0.85	0.75
MBP	-0.34	-0.36	-0.09	-0.16	-0.09	-0.12
MOBP	0.98	0.29	0.79	0.83	0.79	0.80
MOG	0.99	0.32	0.77	0.79	0.75	0.79
OMG	0.85	0.44	0.67	0.77	0.66	0.82
CLDN11	0.98	0.22	0.74	0.73	0.69	0.80
PLP1	0.99	0.30	0.78	0.80	0.76	0.80
POU3F1	-0.15	-0.07	-0.15	0.31	0.32	-0.20
KLK6	0.95	0.27	0.70	0.63	0.59	0.83
EIF2AK3	0.70	0.34	0.55	0.27	0.19	0.66
GAL3ST1	0.96	0.30	0.75	0.74	0.70	0.78
OLIG2	0.94	0.42	0.73	0.78	0.68	0.77
PLLP	0.96	0.38	0.74	0.81	0.75	0.81
NRG1	0.44	0.14	0.57	0.15	0.18	0.29

(b)

Correlation Coefficients across Myelin Gene Expression Vectors (Significant Only)

	CNP	MAG	MAL	MOBP	MOG	CLDN11	PLP1	GAL3ST1	PLLP	ILK	OMG	KLK6	OLIG2
TF	0.97	0.99	0.96	0.98	0.99	0.98	0.99	0.96	0.96	0.85	0.85	0.95	0.94
TFRC	0.29	0.29	0.29	0.29	0.32	0.22	0.3	0.3	0.38	0.18	0.44	0.27	0.42
SLC40A1	0.75	0.77	0.77	0.79	0.77	0.74	0.78	0.75	0.74	0.77	0.67	0.7	0.73
FTH1	0.83	0.75	0.89	0.83	0.79	0.73	0.8	0.74	0.81	0.6	0.77	0.63	0.78
FTL	0.76	0.71	0.85	0.79	0.75	0.69	0.76	0.7	0.75	0.56	0.66	0.59	0.68
SLC11A2	0.82	0.79	0.75	0.8	0.79	0.8	0.8	0.78	0.81	0.75	0.82	0.83	0.77
CNP	1	0.96	0.96	0.97	0.97	0.97	0.98	0.92	0.97	0.85	0.89	0.93	0.94
ILK	0.85	0.84	0.75	0.85	0.82	0.86	0.84	0.82	0.81	1	0.72	0.89	0.8
MAG	0.96	1	0.96	0.97	0.99	0.98	0.99	0.97	0.96	0.84	0.83	0.96	0.94
MAL	0.96	0.96	1	0.97	0.97	0.92	0.97	0.93	0.95	0.75	0.85	0.86	0.94
MBP	-0.29	-0.35	-0.34	-0.32	-0.39	-0.24	-0.35	-0.35	-0.43	-0.09	-0.23	-0.22	-0.42
MOBP	0.97	0.97	0.97	1	0.98	0.96	0.99	0.94	0.96	0.85	0.87	0.93	0.93
MOG	0.97	0.99	0.97	0.98	1	0.97	1	0.96	0.97	0.82	0.86	0.94	0.96
OMG	0.89	0.83	0.85	0.87	0.86	0.86	0.87	0.75	0.88	0.72	1	0.8	0.85
CLDN11	0.97	0.98	0.92	0.96	0.97	1	0.98	0.93	0.93	0.86	0.86	0.97	0.91
PLP1	0.98	0.99	0.97	0.99	1	0.98	1	0.96	0.97	0.84	0.87	0.94	0.95
POU3F1	-0.1	-0.15	0.04	-0.1	-0.1	-0.24	-0.11	-0.07	-0.07	-0.3	-0.27	-0.31	-0.11
KLK6	0.93	0.96	0.86	0.93	0.94	0.97	0.94	0.93	0.9	0.89	0.8	1	0.87
EIF2AK3	0.58	0.69	0.53	0.64	0.64	0.66	0.63	0.7	0.62	0.74	0.51	0.74	0.64
GAL3ST1	0.92	0.97	0.93	0.94	0.96	0.93	0.96	1	0.93	0.82	0.75	0.93	0.93
OLIG2	0.94	0.94	0.94	0.93	0.96	0.91	0.95	0.93	0.95	0.8	0.85	0.87	1
PLLP	0.97	0.96	0.95	0.96	0.97	0.93	0.97	0.93	1	0.81	0.88	0.9	0.95
NRG1	0.44	0.45	0.36	0.38	0.43	0.48	0.44	0.36	0.39	0.53	0.52	**0.47**	0.46
