# Supplementary figures and images for: Quantitative susceptibility mapping in the brain reflects spatial expression of genes involved in iron homeostasis and myelination
Source: Hum Brain Mapp. 2024 Jun 19;45(9):e26688. doi: 10.1002/hbm.26688 (PMC11187871; doi:10.1002/hbm.26688)

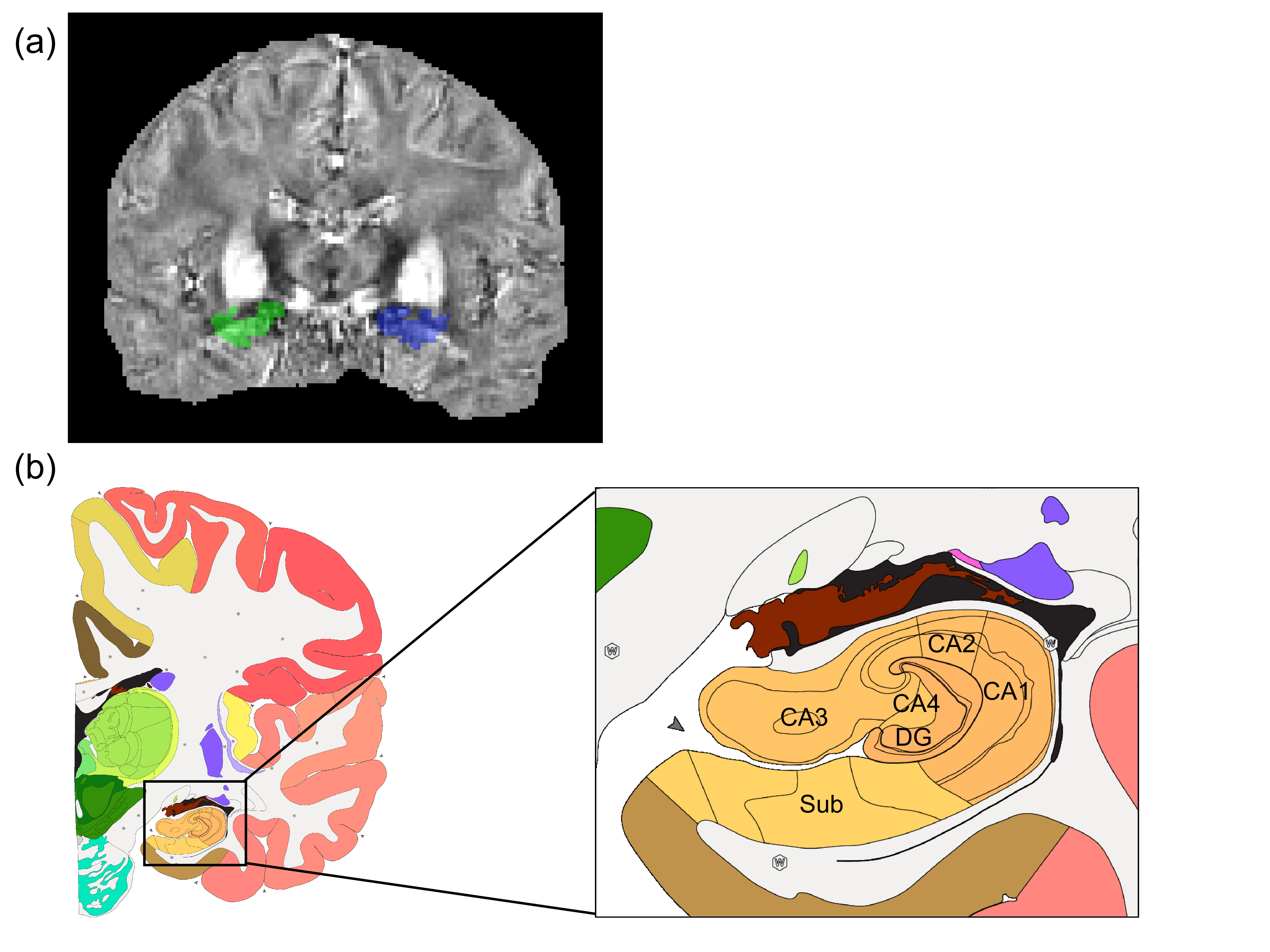

Supplement: Supplementary file 5 — FIGURE S5. Matching QSM and Allen Human Brain Atlas (AHBA) segmentations. In some instances, like the hippocampus, we combined multiple AHBA samples if they fell within the same region, as defined in the QSM segmentation. (a) QSM with hippocampus region segmented. (b) AHBA subdivision of hippocampus (Allen Reference Atlas—Adult Human, human.brain-map.org and atlas.brain-map.org; Ding et al., 2016). Note that the dentate gyrus (DG), CA1 field, CA2 field, CA3 field, CA4 field, and subiculum (Sub) are sampled in the AHBA segmentation, however, we only calculated the average QSM across the entire hippocampus. [file HBM-45-e26688-s007.png]

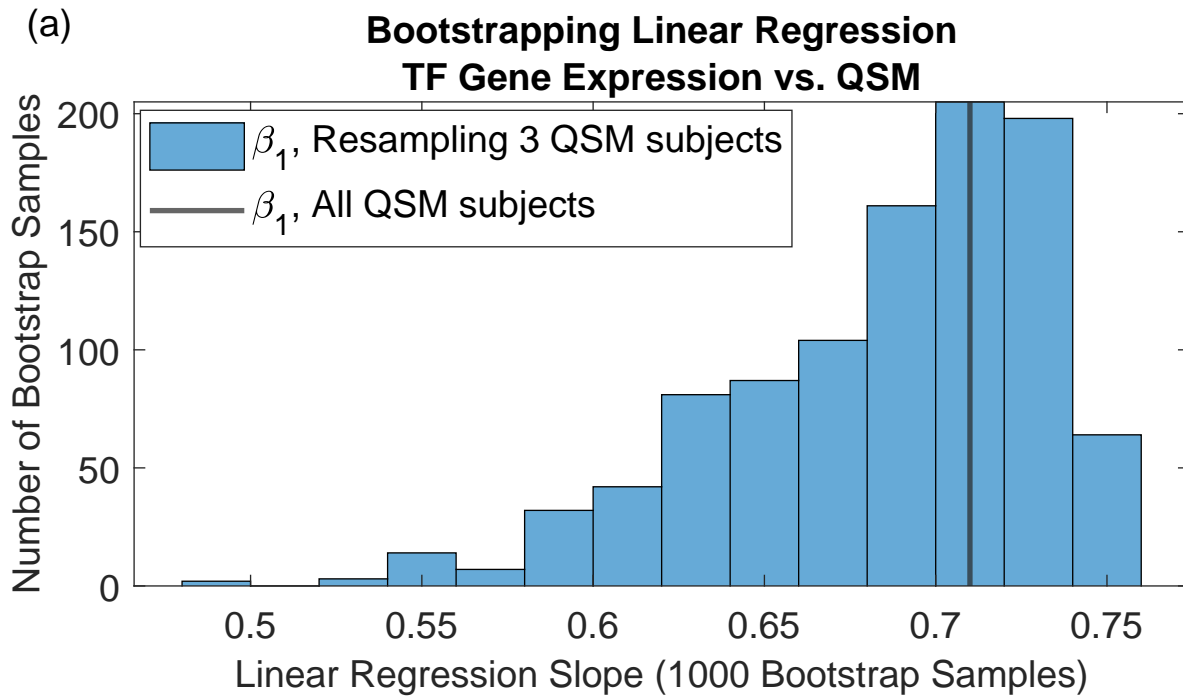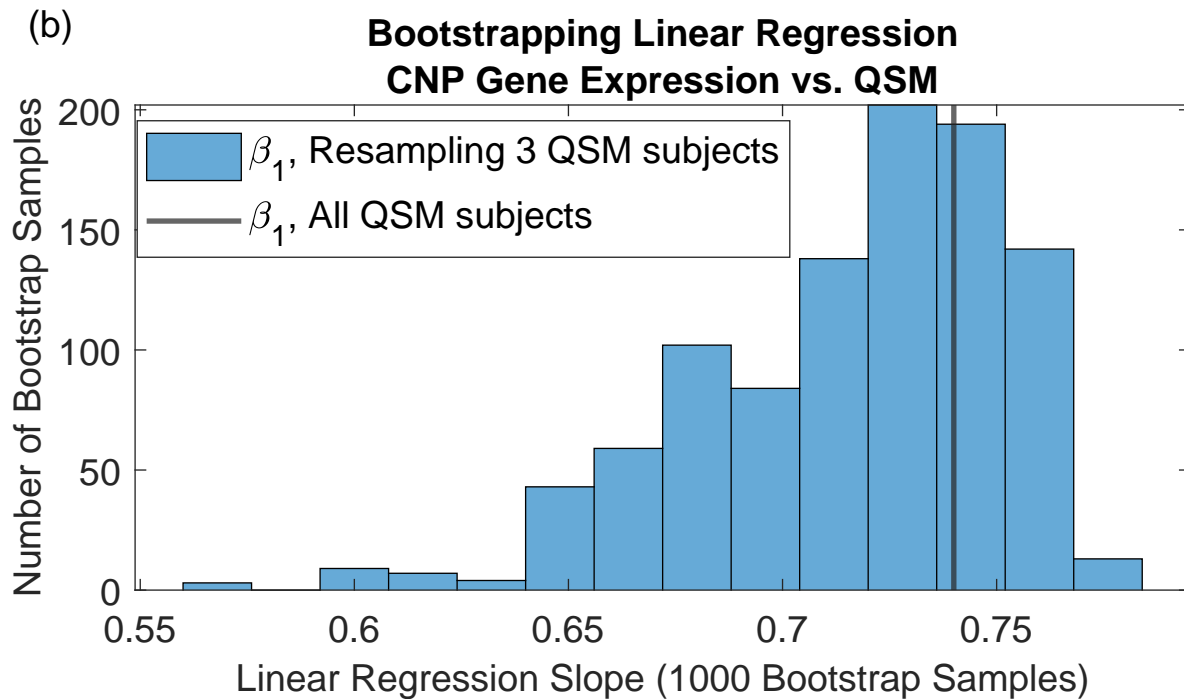

Supplement: Supplementary file 6 — FIGURE S6. Bootstrapping QSM and gene expression linear regression. Resampling three out of the nine QSM subjects, 1st Population, and using the average of only these subjects to perform linear regression with (a) TF gene expression and (b) CNP gene expression. Both results yield distributions with modes that are in close range to the regression coefficient reported using all QSM subjects averaged. [file HBM-45-e26688-s002.pdf]

Gene Expression vs. Avg. QSM Linear Regression  
Deistung et al 2013

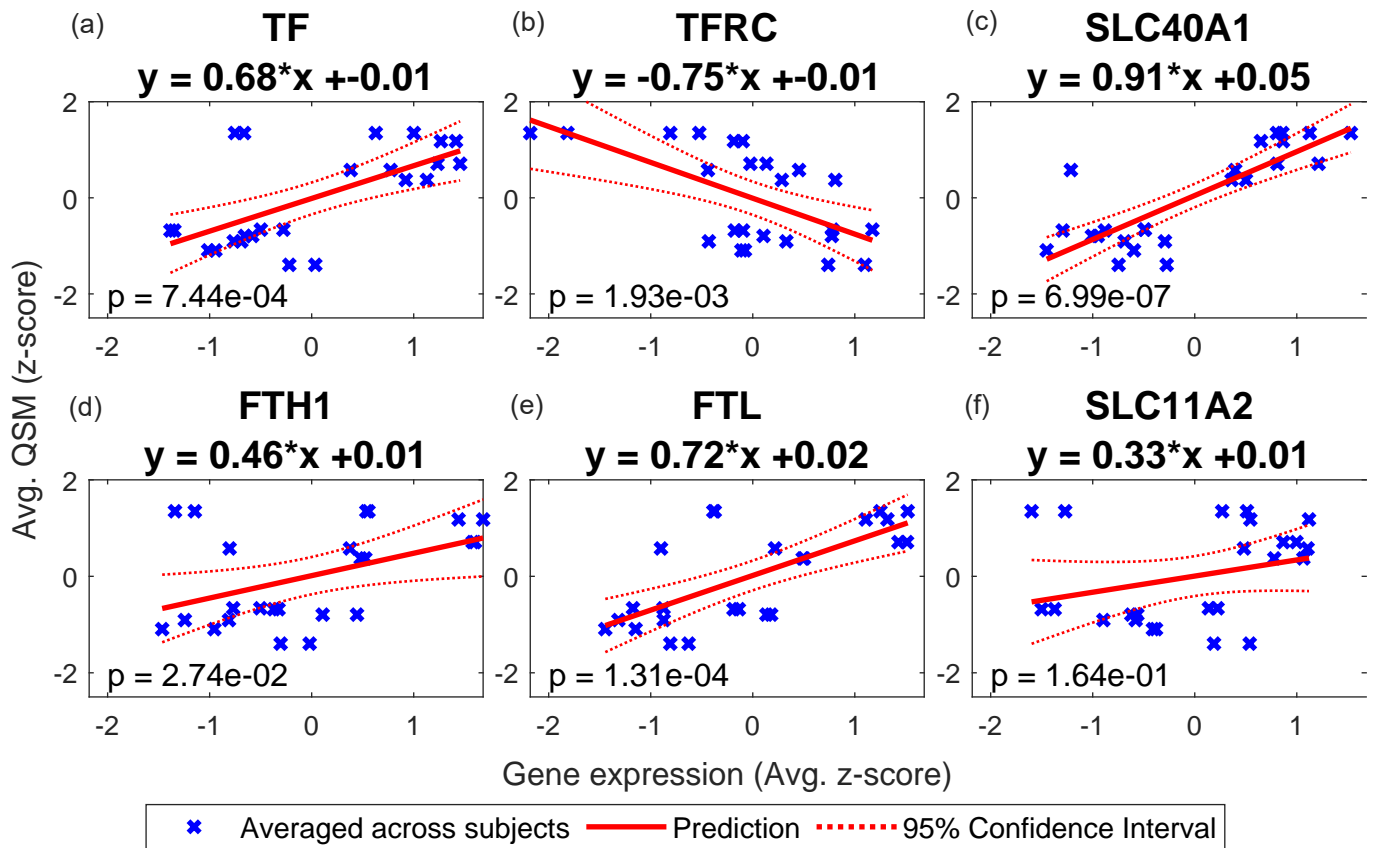

Supplement: Supplementary file 8 — FIGURE S8. Multiple regression of QSM vs. iron related genes, Deistung et al., 2013. Linear regression of QSM from (Deistung et al., 2013) vs. normalized expression of (a) TF, (b) TFRC, (c) SLC40A1, (d) FTH1, (e) FTL, and (f) SLC11A2 in deep grey nuclei regions. These refer to transferrin (TF), transferrin receptor (TFRC), ferroportin (SLC40A1), ferritin heavy chain (FTH1), ferritin light chain (FTL), and divalent metal transporter 1 (SLC11A2). QSM and gene expression were averaged across subjects. Regions of interest in the deep grey nuclei are listed in Figure S7. See Figure S10 for the results of linear regression with the iron gene set performed for each subject separately. [file HBM-45-e26688-s011.pdf]
