# Supplementary material for: Quantitative susceptibility mapping in the brain reflects spatial expression of genes involved in iron homeostasis and myelination
Source: Hum Brain Mapp. 2024 Jun 19;45(9):e26688. doi: 10.1002/hbm.26688 (PMC11187871; doi:10.1002/hbm.26688)
Supplement: Supplementary file 7 — FIGURE S7. Average QSM in Deep Grey Matter Regions, Deistung et al., 2013. (a) Average QSM (b) Normalized average QSM. Regions of interest on the x‐axis correspond to Putamen (Pu), Caudate Nucleus (CN), External Globus Pallidus (ExtGP), Internal Globus Pallidus (IntGP), Substantia Nigra pars reticulata (SNpr), Substantia Nigra pars compacta (SNpc), Red Nucleus (RN), Subthalamic nuclei (SubTl), Anterior nuclei of the Thalamus (AntTl), Median nuclei of the Thalamus (MedTl), Lateral nuclei of the Thalamus (LatTl), and Pulvinar nuclei of the Thalamus (PulTl). These correspond to the mean susceptibility values reported in (Deistung et al., 2013), which are averaged across both hemispheres. The susceptibility values of SNpr and SNpc are the same because these regions aren't distinguished for the value reported for SN in (Deistung et al., 2013). [file HBM-45-e26688-s008.pdf]

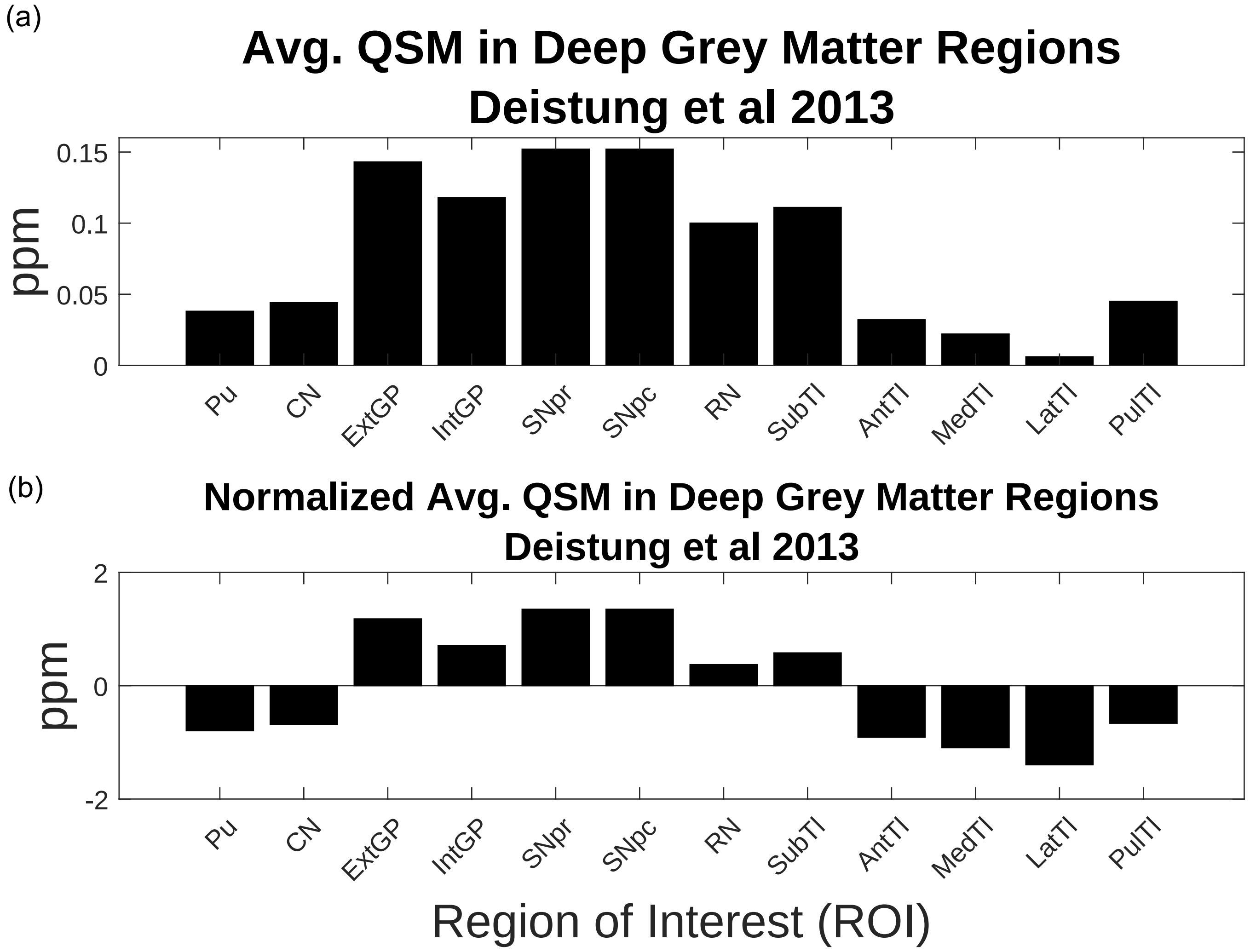
(a)
Avg. QSM in Deep Grey Matter Regions
Deistung et al 2013
ppm
0.15
0.1
0.05
0
Pu
CN
ExtGP
IntGP
SNpr
SNpc
RN
SubTl
AntTl
MedTl
LatTl
PulTl
(b)
Normalized Avg. QSM in Deep Grey Matter Regions
Deistung et al 2013
ppm
2
0
-2
Pu
CN
ExtGP
IntGP
SNpr
SNpc
RN
SubTl
AntTl
MedTl
LatTl
PulTl
Region of Interest (ROI)
