# Supplementary material for: Quantitative susceptibility mapping in the brain reflects spatial expression of genes involved in iron homeostasis and myelination
Source: Hum Brain Mapp. 2024 Jun 19;45(9):e26688. doi: 10.1002/hbm.26688 (PMC11187871; doi:10.1002/hbm.26688)
Supplement: Supplementary file 9 — FIGURE S9. Multiple regression of QSM vs. myelin related genes, Deistung et al., 2013. Linear regression of QSM from (Deistung et al., 2013) vs. normalized expression of (a) CNP, (b) ILK, (c) MAG, (d) MAL, (e) MBP, (f) MOBP, (g) MOG, (h) CLDN11, (i) PLP1, (j) KLK6, (k) GAL3ST1, and (l) PLLP in deep grey nuclei regions. QSM and gene expression were averaged across subjects. Regions of interest in the deep grey nuclei are listed in Figure S7. Only significant results are shown. These include 2′,3′‐cyclic nucleotide 3′‐phosphodiesterase (CNP), integrin‐linked kinase (ILK), myelin‐associated glycoprotein (MAG), myelin and lymphocyte protein (MAL), myelin basic protein (MBP), myelin‐associated oligodendrocytic basic protein (MOBP), myelin oligodendrocyte glycoprotein (MOG), claudin‐11 (CLDN11), proteolipid protein (PLP1), kallikrein‐related peptidase 6 (KLK6), galactose‐3‐O‐sulfotransferase‐1 (GAL3ST1), and proteolipid plasmolipin (PLLP). See Figure S10 for the results of linear regression with the myelin gene set performed for each subject separately. [file HBM-45-e26688-s009.pdf]

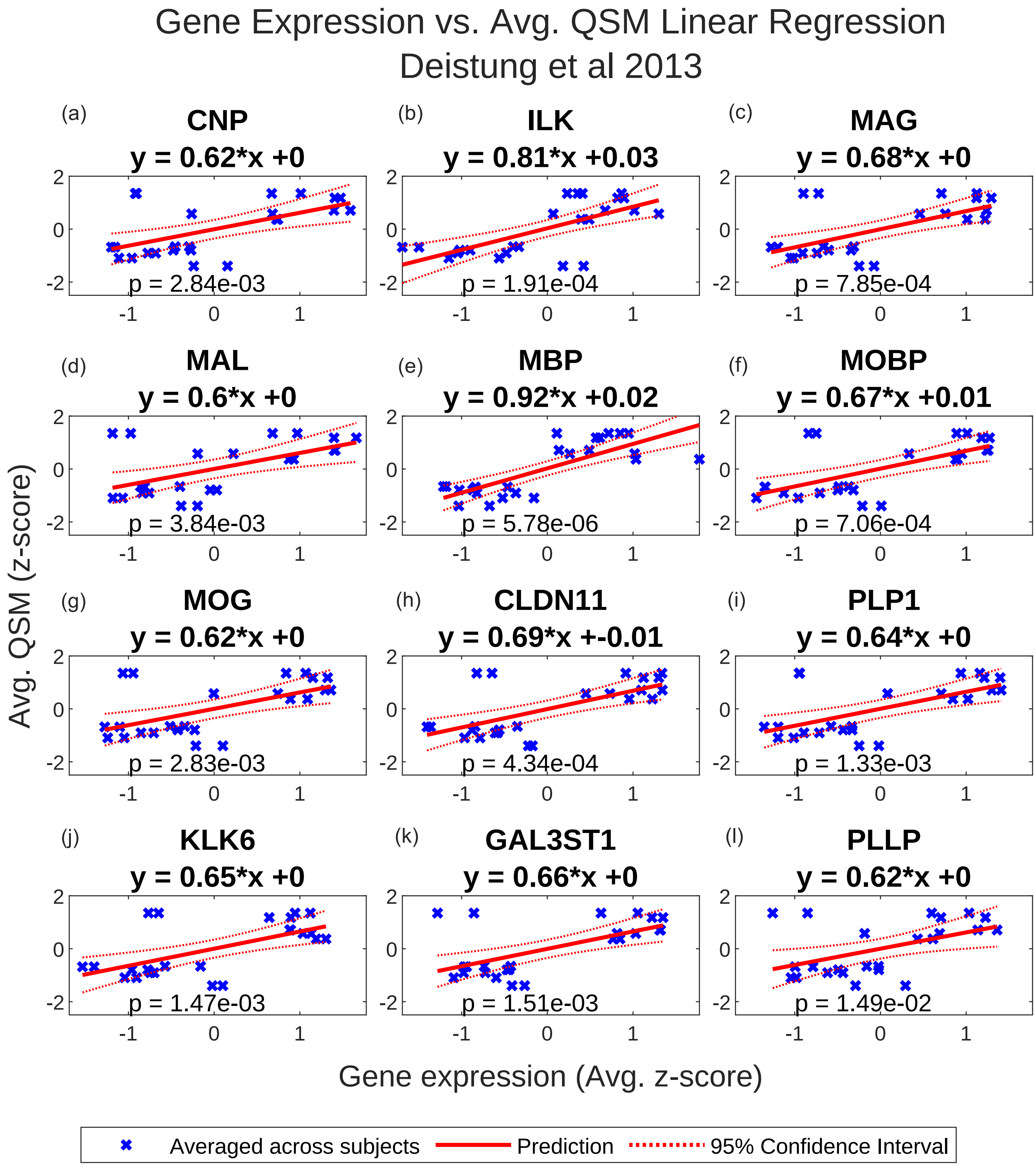

Gene Expression vs. Avg. QSM Linear Regression
Deistung et al 2013
(a) CNP
y = 0.62*x +0
p = 2.84e-03
(b) ILK
y = 0.81*x +0.03
p = 1.91e-04
(c) MAG
y = 0.68*x +0
p = 7.85e-04
(d) MAL
y = 0.6*x +0
p = 3.84e-03
(e) MBP
y = 0.92*x +0.02
p = 5.78e-06
(f) MOBP
y = 0.67*x +0.01
p = 7.06e-04
(g) MOG
y = 0.62*x +0
p = 2.83e-03
(h) CLDN11
y = 0.69*x +-0.01
p = 4.34e-04
(i) PLP1
y = 0.64*x +0
p = 1.33e-03
(j) KLK6
y = 0.65*x +0
p = 1.47e-03
(k) GAL3ST1
y = 0.66*x +0
p = 1.51e-03
(l) PLLP
y = 0.62*x +0
p = 1.49e-02
Avg. QSM (z-score)
Gene expression (Avg. z-score)
Averaged across subjects
Prediction
95% Confidence Interval
