# Supplementary material for: Quantitative susceptibility mapping in the brain reflects spatial expression of genes involved in iron homeostasis and myelination
Source: Hum Brain Mapp. 2024 Jun 19;45(9):e26688. doi: 10.1002/hbm.26688 (PMC11187871; doi:10.1002/hbm.26688)
Supplement: Supplementary file 10 — FIGURE S10. Subject‐level multiple regression of QSM vs. iron and myelin related genes, Deistung et al., 2013. Linear regression of QSM vs. normalized expression of (a) TF, (b) TFRC, (c) SLC11A2, (d) SLC40A1, (e) FTH1, (f) FTL, (g) CNP, (h) MBP, (i) MAL, (j) MOBP, (k) MOG, (l) CLDN11, (m) PLP1, (n) GAL3ST1, (o) PLLP, (p) ILK, (q) KLK6, and (r) MAG in deep grey nuclei regions. These refer to transferrin (TF), transferrin receptor (TFRC), divalent metal transporter 1 (SLC11A2), ferroportin (SLC40A1), ferritin heavy chain (FTH1), ferritin light chain (FTL), 2′,3′‐cyclic nucleotide 3′‐phosphodiesterase (CNP), myelin basic protein (MBP), myelin and lymphocyte protein (MAL), myelin‐associated oligodendrocytic basic protein (MOBP), myelin oligodendrocyte glycoprotein (MOG), claudin‐11 (CLDN11), proteolipid protein (PLP1), galactose‐3‐O‐sulfotransferase‐1 (GAL3ST1), proteolipid plasmolipin (PLLP), integrin‐linked kinase (ILK), kallikrein‐related peptidase 6 (KLK6) and myelin‐associated glycoprotein (MAG). Regressions were performed for each subject in the Allen Human Brain Atlas (AHBA). QSM was averaged across subjects and across regions. Regions of interest in the deep grey nuclei are listed in Figure S7. [file HBM-45-e26688-s003.pdf]

(a) **TF Regression**

Subject	Slope	P-Value
1	0.63	1.04E-03
2	0.45	2.81E-02
3	0.54	7.31E-02
4	0.63	3.89E-02
5	0.70	1.10E-02
6	0.71	9.28E-03

(b) **TFRC Regression**

Subject	Slope	P-Value
1	-0.52	9.59E-03
2	-0.68	2.62E-04
3	-0.07	8.33E-01
4	0.0	9.95E-01
5	-0.58	4.76E-02
6	-0.60	3.82E-02

(c) **SLC11A2 Regression**

Subject	Slope	P-Value
1	0.28	1.91E-01
2	0.07	7.41E-01
3	0.13	6.91E-01
4	0.43	1.87E-01
5	0.54	7.00E-02
6	0.44	1.52E-01

(d) **SLC40A1 Regression**

Subject	Slope	P-Value
1	0.66	4.42E-04
2	0.73	5.73E-05
3	0.76	3.93E-03
4	0.84	1.30E-03
5	0.76	4.11E-03
6	0.94	6.68E-06

(e) **FTH1 Regression**

Subject	Slope	P-Value
1	0.52	8.85E-03
2	0.18	4.01E-01
3	0.43	1.58E-01
4	0.52	9.87E-02
5	0.57	5.44E-02
6	0.62	3.23E-02

(f) **FTL Regression**

Subject	Slope	P-Value
1	0.62	1.10E-03
2	0.63	9.51E-04
3	0.76	4.10E-03
4	0.66	2.59E-02
5	0.67	1.80E-02
6	0.86	3.23E-04

(g) **CNP Regression**

Subject	Slope	P-Value
1	0.57	3.31E-03
2	0.43	3.39E-02
3	0.43	1.67E-01
4	0.62	4.17E-02
5	0.65	2.28E-02
6	0.67	1.80E-02

(h) **MBP Regression**

Subject	Slope	P-Value
1	0.75	2.81E-05
2	0.67	3.08E-04
3	0.56	5.57E-02
4	0.75	7.41E-03
5	0.77	3.37E-03
6	0.69	1.34E-02

(i) **MAL Regression**

Subject	Slope	P-Value
1	0.53	7.53E-03
2	0.35	9.36E-02
3	0.51	9.39E-02
4	0.62	4.28E-02
5	0.64	2.45E-02
6	0.72	8.65E-03

(j) **MOBP Regression**

Subject	Slope	P-Value
1	0.67	3.63E-04
2	0.47	2.11E-02
3	0.62	3.02E-02
4	0.67	2.55E-02
5	0.63	2.67E-02
6	0.75	4.57E-03

(k) **MOG Regression**

Subject	Slope	P-Value
1	0.58	3.00E-03
2	0.41	4.40E-02
3	0.33	2.92E-01
4	0.58	5.94E-02
5	0.69	1.38E-02
6	0.73	6.69E-03

(l) **CLDN11 Regression**

Subject	Slope	P-Value
1	0.58	2.80E-03
2	0.60	2.07E-03
3	0.46	1.28E-01
4	0.68	2.08E-02
5	0.65	2.29E-02
6	0.67	1.61E-02

(m) **PLP1 Regression**

Subject	Slope	P-Value
1	0.64	7.32E-04
2	0.46	2.38E-02
3	0.51	8.96E-02
4	0.60	5.14E-02
5	0.67	1.63E-02
6	0.68	1.54E-02

(n) **GAL3ST1 Regression**

Subject	Slope	P-Value
1	0.57	3.56E-03
2	0.41	4.70E-02
3	0.36	2.47E-01
4	0.63	3.97E-02
5	0.57	5.21E-02
6	0.71	9.51E-03

(o) **PLLP Regression**

Subject	Slope	P-Value
1	0.51	1.06E-02
2	0.13	5.60E-01
3	0.14	6.57E-01
4	0.41	2.06E-01
5	0.59	4.43E-02
6	0.68	1.54E-02

(p) **ILK Regression**

Subject	Slope	P-Value
1	0.56	4.03E-03
2	0.44	3.15E-02
3	0.74	5.77E-03
4	0.73	1.00E-02
5	0.45	1.42E-01
6	0.79	2.27E-03

(q) **KLK6 Regression**

Subject	Slope	P-Value
1	0.59	2.51E-03
2	0.51	1.09E-02
3	0.46	1.29E-01
4	0.64	3.40E-02
5	0.61	3.59E-02
6	0.66	1.94E-02

(r) **MAG Regression**

Subject	Slope	P-Value
1	0.58	3.01E-03
2	0.49	1.45E-02
3	0.39	2.12E-01
4	0.65	2.90E-02
5	0.65	2.23E-02
6	0.76	4.08E-03
