# Supplementary material for: Quantitative susceptibility mapping in the brain reflects spatial expression of genes involved in iron homeostasis and myelination
Source: Hum Brain Mapp. 2024 Jun 19;45(9):e26688. doi: 10.1002/hbm.26688 (PMC11187871; doi:10.1002/hbm.26688)
Supplement: Supplementary file 11 — TABLE S1. Full set of myelination related genes used in linear regression analysis. The regression results were reported only for myelin genes significantly correlated with QSM, following the Benjamini–Hochburg procedure. The abbreviation for each gene and the corresponding protein encoded by the gene are listed in the table above. [file HBM-45-e26688-s006.docx]

| **Myelination Gene Set** | |
| --- | --- |
| **Gene** | **Protein** |
| CLDN11 | Claudin11 |
| GAL3ST1 | Galactose-3-O-sulfotransferase-1 |
| MAG | Myelin-associated glycoprotein |
| OMG | Oligodendrocyte myelin glycoprotein |
| MBP | Myelin basic protein |
| CNP | 2’,3’-cyclic nucleotide 3’-phosphodiesterase |
| ILK | Integrin-linked kinase |
| MAL | Myelin and lymphocyte protein |
| PLLP | Proteolipid plasmolipin |
| NRG1 | Neuregulin 1 |
| EIF2AK3 | Eukaryotic translation initiation factor 2 alpha kinase 3 |
| KLK6 | Kallikrein-6 |
| PLP1 | Proteolipid protein |
| POU3F1 | POU domain, class 3, transcription factor 1 |
| OLIG2 | Oligodendrocyte transcription factor 2 |
| MOBP | Myelin-associated oligodendrocytic basic protein |
| MOG | Myelin oligodendrocyte glycoprotein |
