# Supplementary material for: Quantitative susceptibility mapping in the brain reflects spatial expression of genes involved in iron homeostasis and myelination
Source: Hum Brain Mapp. 2024 Jun 19;45(9):e26688. doi: 10.1002/hbm.26688 (PMC11187871; doi:10.1002/hbm.26688)
Supplement: Supplementary file 12 — TABLE S2. Full set of iron homeostasis genes used in linear regression analysis. The regression results were reported for all of these genes. The abbreviation for each gene and the corresponding protein encoded by the gene are listed in the table above. [file HBM-45-e26688-s004.docx]

| **Iron Gene Set** | |
| --- | --- |
| **Gene** | **Protein** |
| TF | Transferrin (Tf) |
| TFRC | Transferrin receptor (TFR) |
| SLC11A2 | Divalent metal transporter 1 (DTM1) |
| FTL | Light-chain ferritin (L-Ft) |
| FTH1 | Heavy-chain ferritin (H-Ft) |
| SLC40A1 | Ferroportin (Fpn) |
